# Exploiting 4-1BB immune checkpoint to enhance the efficacy of oncolytic virotherapy for diffuse intrinsic pontine gliomas

**DOI:** 10.1172/jci.insight.154812

**Published:** 2022-04-08

**Authors:** Virginia Laspidea, Montserrat Puigdelloses, Sara Labiano, Lucía Marrodán, Marc Garcia-Moure, Marta Zalacain, Marisol Gonzalez-Huarriz, Naiara Martínez-Vélez, Iker Ausejo-Mauleon, Daniel de la Nava, Guillermo Herrador-Cañete, Javier Marco-Sanz, Elisabeth Guruceaga, Carlos E. de Andrea, María Villalba, Oren Becher, Massimo Squatrito, Verónica Matía, Jaime Gállego Pérez-Larraya, Ana Patiño-García, Sumit Gupta, Candelaria Gomez-Manzano, Juan Fueyo, Marta M. Alonso

**Affiliations:** 1 Health Research Institute of Navarra, Pamplona, Navarra, Spain.; 2 Solid Tumor Program, Center for the Applied Medical Research, Pamplona, Navarra, Spain.; 3 Department of Pediatrics, Navarra University Clinic, Pamplona, Spain.; 4 Gene Therapy and Regulation of Gene Expression Program, Center for the Applied Medical Research, Pamplona, Navarra, Spain.; 5 Bioinformatics Platform, El Centro de Investigación Médica Aplicada (CIMA), University of Navarra, Pamplona, Spain.; 6 Department of Pathology, Navarra University Clinic, Pamplona, Spain.; 7 Department of Pediatrics,; 8 Department of Biochemistry and Molecular Genetics, and; 9 Lurie Comprehensive Cancer Center, Northwestern University, Chicago, Illinois, USA.; 10 Division of Hematology Oncology and Stem Cell Transplant, Ann & Robert H. Lurie Children’s Hospital, Chicago, Illinois, USA.; 11 Seve Ballesteros Foundation Brain Tumor Group, Molecular Oncology Programme, Spanish National Cancer Research Center, Madrid, Spain.; 12 Department of Neurology, Navarra University Clinic, Pamplona, Spain.; 13 Department of Neuro-Oncology, The University of Texas MD Anderson Cancer Center, Houston, Texas, USA.

**Keywords:** Immunology, Oncology, Brain cancer, Cancer immunotherapy, Immunotherapy

## Abstract

Diffuse intrinsic pontine gliomas (DIPGs) are aggressive pediatric brain tumors, and patient survival has not changed despite many therapeutic efforts, emphasizing the urgent need for effective treatments. Here, we evaluated the anti-DIPG effect of the oncolytic adenovirus Delta-24-ACT, which was engineered to express the costimulatory ligand 4-1BBL to potentiate the antitumor immune response of the virus. Delta-24-ACT induced the expression of functional 4-1BBL on the membranes of infected DIPG cells, which enhanced the costimulation of CD8^+^ T lymphocytes. In vivo, Delta-24-ACT treatment of murine DIPG orthotopic tumors significantly improved the survival of treated mice, leading to long-term survivors that developed immunological memory against these tumors. In addition, Delta-24-ACT was safe and caused no local or systemic toxicity. Mechanistic studies showed that Delta-24-ACT modulated the tumor-immune content, not only increasing the number, but also improving the functionality of immune cells. All of these data highlight the safety and potential therapeutic benefit of Delta-24-ACT the treatment of patients with DIPG.

## Graphical abstract

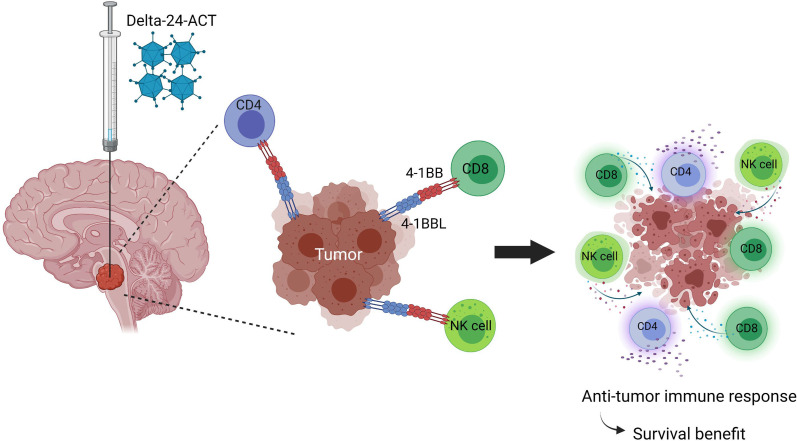

## Introduction

Diffuse intrinsic pontine gliomas (DIPGs) are aggressive pediatric brain stem tumors that most frequently occur in children ranging in age from 6 to 9 years. With a median overall survival of 9–11 months, DIPGs are the main cause of cancer-related death in children (1, 2). In 2016, the WHO reclassified CNS tumors and included DIPG within a new class: H3-K27M mutant diffuse midline glioma (DMG H3-K27M) (3). Approximately 80% of DIPGs contain H3-K27M mutations (usually in histone H3.3 or H3.1), which have been proposed as driver mutations in this malignancy (4). Other relevant mutations include *TP53* (40%–77%), *PDGFR* (13%–36%), and *ACVR1* (20%–32%) (5, 6).

Because of the disseminating nature and localization of the tumor, surgical resection is not an option, and the current standard treatment is radiotherapy, which slightly improves the survival and quality of life of patients with DIPG in the short term. However, less than 10% of patients survive for more than 2 years after diagnosis (7–9), pointing to the urgent need for effective therapies. DIPGs are immunologically “cold” tumors due to their low mutational burden and extremely low levels of immune infiltration (10). Recent studies demonstrated that the DIPG microenvironment is mainly made up of microglia/macrophages and that T cell infiltration is minimal (10–12). Because T cells constitute an essential part of the antitumor effect in immune therapies (13), strategies geared toward increasing cytotoxic T cell infiltration are desirable to trigger an effective antitumor immune response, and one such strategy is based on the use of oncolytic adenoviruses (14). Oncolytic viruses are tumor-selective biotherapeutic agents that promote antitumor responses via the cytolysis of tumor cells and the induction of tumor-specific immune responses engaging the innate and adaptive arms. We previously demonstrated that the treatment of DIPG and pediatric high-grade glioma human and murine models with the oncolytic virus Delta-24-RGD increased the survival rates; however, not all of our study animals were cured, indicating that therapeutic improvement is possible (15, 16). Moreover, a phase I clinical trial of DNX-2401 (Delta-24-RGD) in patients with glioblastoma demonstrated that the administered virus was safe and improved patient survival by promoting an increase in tumor immune infiltration (17). In this study, we explored the idea of combining the cytotoxic effect of Delta-24-RGD with the activation of the costimulatory immune checkpoint 4-1BB to further boost the immune effect of the virus. The 4-1BB (also known as CD137 and TNFRSF9) protein is a member of the TNF receptor superfamily that is expressed by activated T lymphocytes and NK cells, among other cell types. Its ligand, 4-1BBL (also known as CD137L), is expressed on antigen-presenting cells, such as DCs, macrophages, and B cells (18). The binding of 4-1BB to its ligand promotes T cell activation in both CD4^+^ and CD8^+^ lymphocytes (19). The targeting of 4-1BB, especially by agonist monoclonal antibodies, has yielded promising results in solid tumors (20, 21). However, the systemic administration of such antibodies has been associated with liver toxicity (20). Therefore, we genetically modified Delta-24-RGD to express the costimulatory ligand of 4-1BB (4-1BBL), generating a new virus, Delta-24-ACT (22), for the delivery of 4-1BBL directly into the tumor. This virus displayed antitumor effects in murine glioma models and demonstrated a safe profile. In this study, we characterized the oncolytic capacity of Delta-24-ACT and its ability to recruit immune cells in DIPG. Our results showed that Delta-24-ACT produced a functional 4-1BBL that activated immune cell effector and memory functions in preclinical DIPG models and exhibited a therapeutic effect superior to that of parental Delta-24-RGD while maintaining a safe profile.

## Results

### 4-1BBL expressed by Delta-24-ACT costimulates T cells.

**Figure 1:**
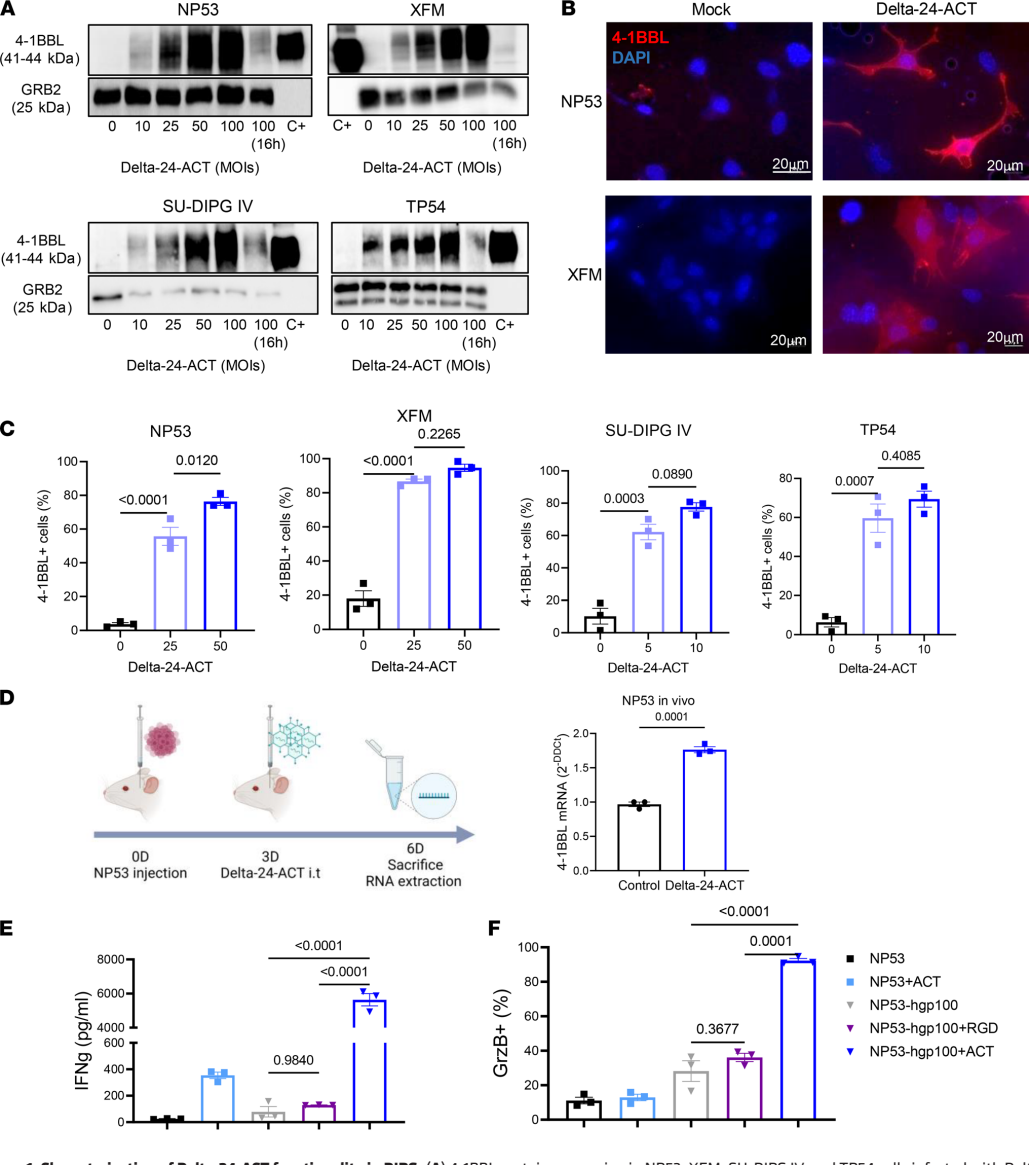
Characterization of Delta-24-ACT functionality in DIPG. (**A**) 4-1BBL protein expression in NP53, XFM, SU-DIPG IV, and TP54 cells infected with Delta-24-ACT at the indicated MOIs, as determined by Western blotting. C+, 4-1BBL recombinant protein. (**B**) Representative immunofluorescence images of 4-1BBL expression in NP53- and XFM-infected cells compared with mock-infected cells. Scale bar: 20 μm. (**C**) 4-1BBL protein expression in the membranes of murine and human cells infected with Delta-24-ACT at the indicated MOIs, as determined by flow cytometry. The percentage of 4-1BBL^+^ cells is shown. One-way ANOVA was performed (*n* = 3, each group), and *P* values are shown above respective bars. Data are shown as the mean ± SEM. (**D**) Schedule of the experiment for the in vivo determination of 4-1BBL expression, and evaluation of 4-1BBL protein expression in NP53 tumors from control- or Delta-24-ACT–treated mice (*n* = 3), as determined by Q-PCR. Data are shown as the mean ± SEM. (**E**) IFN-γ and (**F**) granzyme B production by CD8^+^ lymphocytes. CD8^+^ T cells from PMEL mice were cocultured with NP53 cells infected with Delta-24-RGD, Delta-24-ACT (MOI = 100), or the mock control. CD8^+^ lymphocytes activated with CD3 and CD28 but not NP53 cells, and CD8^+^ lymphocytes activated with CD3, CD28, and 4-1BB antibody were used as negative and positive controls for the experiment, respectively. One-way ANOVA was performed (*n* = 3, each group), and *P* values are shown above respective bars. Data are shown as the mean ± SEM.

In this work, we aimed to improve the ability of Delta-24-RGD to generate an effective antitumor immune response in DIPG tumors. To do so, Delta-24-RGD was genetically modified by incorporation of a cassette expressing the murine 4-1BBL in place of the E3 gene to generate a new oncolytic adenovirus, Delta-24-ACT (22) (Supplemental Figure 1A; supplemental material available online with this article; https://doi.org/10.1172/jci.insight.154812DS1). Delta-24-ACT maintains the same genetic modifications as Delta-24-RGD (23), a 24–base pair deletion in E1A and the introduction of RGD-4C in the HI loop fiber. After generation of the virus, we first determined its ability to express the ligand in DIPG murine (NP53 and XFM) and human (SU-DIPG IV and TP54) cell lines after infection. We readily detected 4-1BBL protein (Figure 1A) and mRNA (Supplemental Figure 1B) expression in infected murine and human cell lines in a dose-dependent manner. More importantly, we detected 4-1BBL expression on the membranes of infected cells, which is essential for correct functioning, by immunofluorescence and flow cytometry (Figure 1, B and C, and Supplemental Figure 1C). In fact, 4-1BBL expression was detected in 80% of infected mouse cell membranes at a multiplicity of infection (MOI) of 50, whereas this percentage was reached at an MOI of 10 on the surface of human cell membranes (Figure 1C). Of importance, we also observed a significant increase in the expression of the ligand in mouse DIPG tumors treated with Delta-24-ACT in vivo (Figure 1D). Moreover, functional studies in vitro demonstrated that the 4-1BBL expressed by the virus was functional and capable of stimulating CD8^+^ lymphocytes. We performed an experiment in which gp100-specific CD8^+^ T cells from PMEL mice were cocultured with mock-, Delta-24-RGD–, or Delta-24-ACT–infected NP53 cells (that were previously pulsed with the gp100 peptide) for 48 hours. We observed a significant increase in IFN-γ and granzyme B expression in T lymphocytes cocultured with NP53 cells infected with Delta-24-ACT compared with those cocultured in the presence of noninfected or Delta-24-RGD–infected NP53 cells (Figure 1, E and F). In fact, activated CD8^+^ T lymphocytes cocultured with Delta-24-ACT–infected NP53 cells acquired a more blastic morphology and formed more clusters in vitro (Supplemental Figure 1D).

### Delta-24-ACT maintains the oncolytic features of Delta-24-RGD and induces immunogenic cell death in vitro.

**Figure 2:**
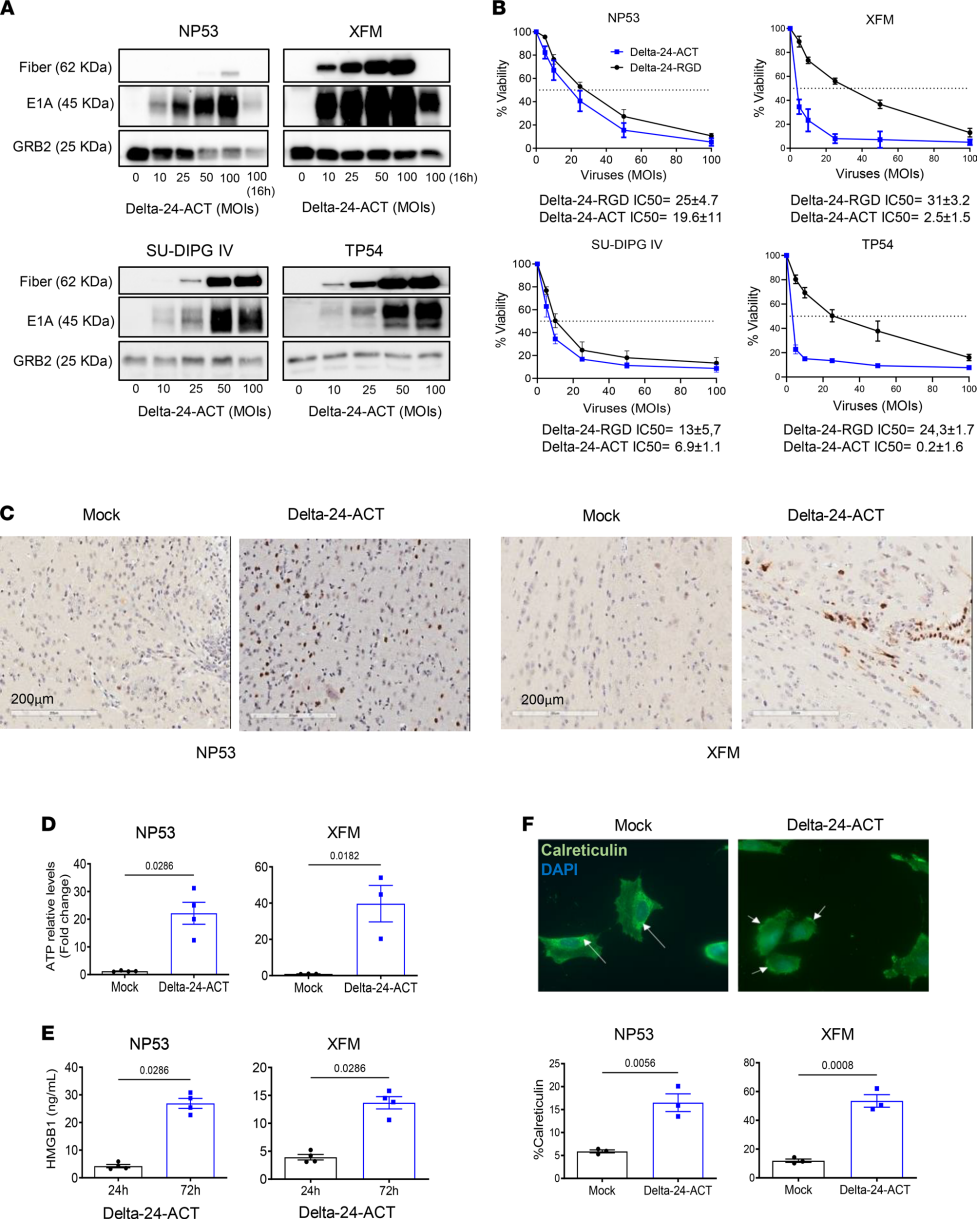
Evaluation of the Delta-24-ACT oncolytic effects in vitro. (**A**) Assessment of viral protein expression (fiber and E1A) in NP53, XFM, SU-DIPG IV, and TP54 cells by Western blotting. Cells were infected with Delta-24-ACT at the indicated MOIs, and whole-cell lysates were collected 48 hours later. GRB2 was used as a protein-loading control. (**B**) Oncolytic effects of Delta-24-ACT on murine and human DIPG cells. To quantify the oncolytic effects of Delta-24-RGD or Delta-24-ACT on cells, they were infected at the indicated MOIs, and viability was evaluated 5 days later by MTS assays. Values indicate the percentages of viable cells in infected cultures compared with noninfected cultures (mean ± SD, *n* = 3 each group). (**C**) Evaluation of the E1A viral protein in vivo. Viral protein expression was evaluated in vivo in mice bearing either XFM or NP53 cells 3 days after mock or Delta-24-ACT treatment. Representative micrographs are shown. Scale bar: 20 μm. (**D** and **E**) Concentrations of the damage-associated molecular pattern (DAMP) markers ATP and HMGB1 in supernatants obtained from NP53 and XFM cultures at 72 hours after Delta-ACT (*n* = 4) or mock (*n* = 4) infection. Data are shown as the mean ± SEM (Mann-Whitney test), and *P* values are shown above bars. (**F**) Representative fluorescence microscopy images of NP53 cells at 4 hours after infection with Delta-24-ACT or mock infection. Calreticulin (CRT) at the cell surface was detected by immunofluorescence (green) and nuclei (blue; DAPI). Arrows denote the calreticulin location in the cell. Original magnification, ×40. Flow cytometric quantification of membrane calreticulin^+^ cells after Delta-24-ACT infection. Data are shown as the mean ± SEM (*n* = 4 each group; Mann-Whitney test), and *P* values are shown above bars.

Once we confirmed that 4-1BBL was functional, we next characterized the oncolytic effect of Delta-24-ACT in mouse and human DIPG cell lines. Western blotting was used to analyze E1A expression and adenovirus fiber expression, which are indicative of infection and viral replication, respectively, and we observed dose-dependent changes in viral protein expression in all the cell lines tested (Figure 2A). Next, we tested the cytotoxic effect of Delta-24-ACT 5 days after viral infection (MOIs ranging from 5 to 100) and compared it with that of Delta-24-RGD. We observed that the percentage of viable cells decreased as the viral input increased with both viruses. Delta-24-ACT displayed a half-maximal inhibitory concentration (IC_50_) ranging from an MOI of 16.1 to an MOI of 0.2; the values were lower in the human DIPG cell lines than in mouse cell lines as a result of effective replication (NP53, 16.1 ± 1.1 PFU/cell; SU-DIPG IV, 6.9 ± 1.1 PFU/cell; XFM, 2.5 ± 1.5 PFU/cell; and TP54, 0.2 ± 1.6 PFU/cell; Figure 2B). Delta-24-RGD showed similar IC_50_ values in vitro, as expected. E1A expression observed by immunohistochemistry in NP53- and XFM-bearing mice treated with Delta-24-ACT 4 days after viral administration (Figure 2C) indicated that the virus can infect tumor cells in vivo.

Immunogenic cell death is accompanied by the exposure and release of damage-associated molecular patterns (DAMPs), which ultimately favor an immune response. Importantly, we observed significant increases in secreted ATP and HMGB1 levels following Delta-24-ACT infection of NP53 and XFM cells (Figure 2, D and E); ATP and HMGB1 are well-described DAMPs (24). Delta-24-ACT infection triggered the translocation of calreticulin to the plasma membrane (Figure 2F), another well-known mechanism that contributes to the immunogenicity of tumor cells by acting as an “eat-me” signal to promote tumor cell phagocytosis by macrophages (25). In summary, Delta-24-ACT was shown to infect murine and human DIPG cell lines, to express functional 4-1BBL, and to exert oncolytic effects leading to the triggering of several DAMPs associated with immunogenic cell death.

### Delta-24-ACT exhibits a safe profile in vivo.

**Figure 3:**
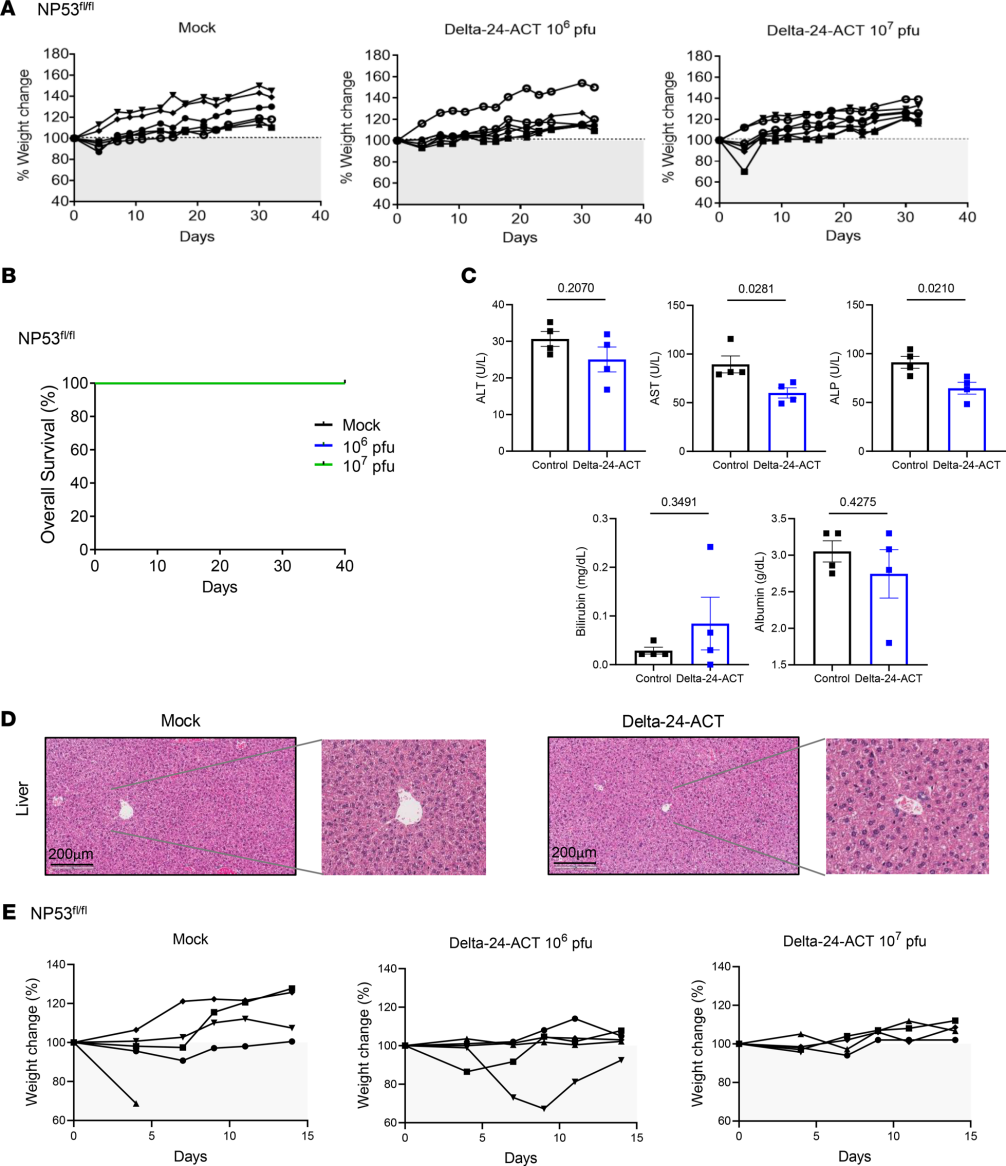
Assessment of Delta-24-ACT toxicity in vivo. (**A**) NP53^fl/fl^ mice were treated intraparenchymally with mock treatment (PBS) (*n* = 6) or Delta-24-ACT (*n* = 7) at the indicated doses. Mice from the different groups were weighed every 3–4 days until the end of the treatment (30 days). (**B**) Kaplan-Meier survival plot of NP53^fl/^ mice treated with PBS (control group) and 10^6^ PFUs or 10^7^ PFUs of Delta-24-ACT in the pons. (**C**) Evaluation of biochemical parameters related to hepatic toxicity after intratumoral injection of Delta-24-ACT. The mice were treated with the mock treatment or virus, and serum samples were collected 3 days later. Several parameters were measured, including alanine aminotransferase (ALT, U/L), aspartate aminotransferase (AST, U/L), and alkaline phosphatase (ALP, U/L) levels, to monitor hepatic injury and bilirubin (mg/dL) and albumin (g/dL) levels to assess hepatic function. Student’s *t* test was performed, and *P* values are shown above bars. Data are shown as the mean ± SEM. (**D**) Histologic analysis of mouse livers bearing orthotopic DIPGs and treated locally with Delta-24-ACT at 10^8^ PFUs. Representative micrographs of H&E staining of mouse livers from the indicated groups of DIPG models. Scale bar: 200 μm. The images show no viral presence in mouse livers and no signs of hepatotoxicity. (**E**) Percentage of weight change in NP53^fl/fl^ mice bearing NP53 tumors treated with 10^6^ or 10^7^ PFUs/mouse Delta-24-ACT or PBS (as a control).

The administration of an oncolytic virus with the capacity to unleash a potent immune response could result in lethal inflammation of the brain stem. In addition, 4-1BB agonist antibody administration in the clinic has been associated with liver toxicity (20). Thus, we first studied the safety profile of Delta-24-ACT in vivo by either administering different doses of the virus (10^6^ and 10^7^ PFUs/mouse) or PBS as a control into the pons of 2 tumor-free immunocompetent mouse strains (NP53^fl/fl^ and BALB/c). We assessed whether the virus had any effect in healthy tissue before viral administration into tumor-bearing mice. We weighed the animals every 2–3 days to assess weight variability as a sign of toxicity and observed no significant weight loss at any of the viral concentrations evaluated (Figure 3A and Supplemental Figure 2A). We observed no deaths among any of the NP53^fl/fl^ strain groups (Figure 3B). Among BALB/c mice, there was 1 death each in the PBS and 10^6^ PFU groups within the first 7 days of treatment (Supplemental Figure 2B). Evaluation of the brains ruled out lethal inflammation, and we believe that these deaths were due to the procedure itself. The absence of a gross loss of body weight suggested that the viral injection was well tolerated. In a separate experiment, we obtained sera from mice bearing NP53 cells treated with either Delta-24-ACT or PBS (mock control) and measured several parameters, including alanine aminotransferase (ALT), aspartate aminotransferase (AST), and alkaline phosphatase (ALP) levels to monitor hepatic injury and bilirubin and albumin levels to monitor hepatic function. We observed no significant differences between the virus-treated and PBS-treated mice in the measured parameters, except for AST and ALP levels, which were significantly increased in control- (PBS-treated) mice. However, the obtained values were within the range of normal values in mice (AST = 50–100 U/L, ALP = 35–100 U/L) (Figure 3C). To further analyze hepatic toxicity, we conducted anatomopathological analyses of mouse livers 15 days after viral administration to detect histological changes in this organ induced by Delta-24-ACT. Importantly, none of the analyzed livers showed signs of hepatic injury (Figure 3D and Supplemental Figure 2C). Moreover, we did not observe significant weight loss in NP53-bearing mice in the days after the administration of different doses of the virus (10^6^ and 10^7^ PFU/mouse) or PBS (Figure 3E). These results highlight the safety of Delta-24-ACT and the fact that intratumoral injection of 4-1BBL does not induce hepatic toxicity.

### Delta-24-ACT increased survival and promoted immunological memory in orthotopic DIPG models.

**Figure 4:**
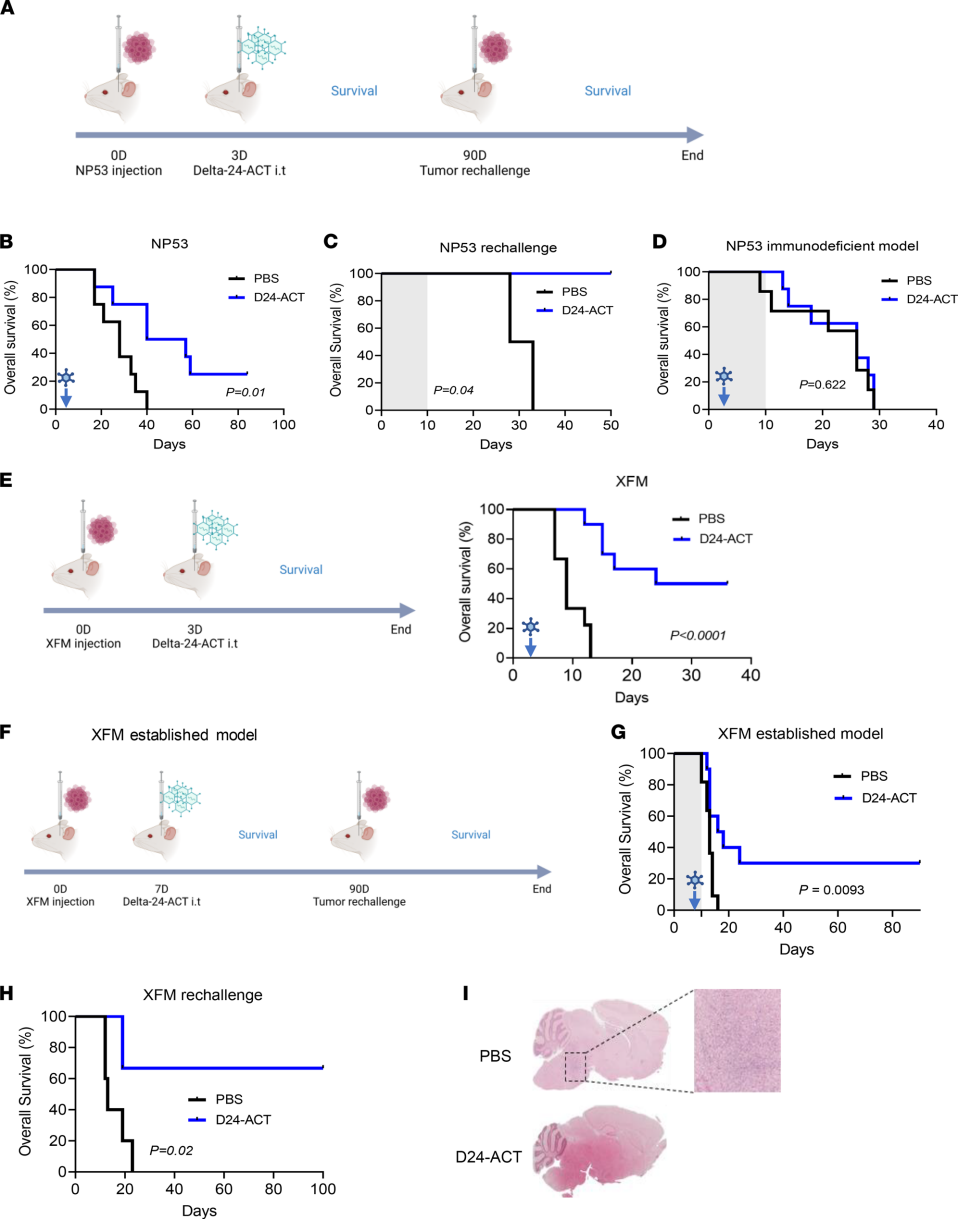
Characterization of the antitumor effect of Delta-24-ACT in DIPG orthotopic models. (**A**) Schedule of survival experiments performed with NP53 cells. (**B**) Kaplan-Meier survival plot of mice bearing NP53 cells treated with 10^6^ PFUs of Delta-24-ACT or a mock PBS control 3 days after cell administration (log-rank; *P* = 0.01, *n* = 8 each group). (**C**) The long-term survivors from the Delta-24-ACT–treated group (*n* = 2) were subjected to rechallenge with NP53 and compared with control naive mice (*n* = 4) (log-rank; *P* < 0.04). (**D**) Kaplan-Meier survival plot of immunodeficient mice (BALB/cA-Rag2^−/−^γc^−/−^) bearing NP53 cells treated with 10^6^ PFUs of Delta-24-ACT (*n* = 8) or a mock PBS control (*n* = 7) 3 days after cell administration (log-rank; *P* = 0.600). (**E**) Schedule of survival experiments performed with XFM cells, and Kaplan-Meier survival plot of mice bearing XFM cells that were treated with 10^6^ PFUs of Delta-24-ACT (*n* = 10) or the mock PBS control 3 days after cell administration (*n* = 9) (log-rank; *P* < 0.0001). (**F**) Schedule of the survival experiment with the established XFM model; Delta-24-ACT was administered 7 days after cell injection. (**G**) Kaplan-Meier survival plot of mice bearing XFM-established tumors treated with 10^6^ PFUs of Delta-24-ACT (*n* = 10) or a mock PBS control (*n* = 11) 7 days after cell administration (log-rank; *P* = 0.0009). (**H**) The long-term survivors from the Delta-24-ACT–treated group (*n* = 3) were subjected to rechallenge with XFM and compared with control naive mice (*n* = 5) (log-rank; *P* = 0.02). (**I**) Representative micrographs of XFM long-term survivors free of disease versus a naive control (PBS) that presented a tumor. Original magnification, ×4; ×100 (high-magnification image).

To evaluate the anti-DIPG effect of Delta-24-ACT in vivo, DIPG tumors were developed by the intracranial injection of NP53 or XFM cells into the pons of immunocompetent mice (26). Three days after cell injections, mice were treated with PBS (control group) or Delta-24-ACT (10^6^ PFUs/mouse) and were monitored for survival (Figure 4A). We observed a significant increase in the median survival of mice treated with the virus in comparison to the control groups in both models (log-rank test; *P* = 0.014 for NP53 cells, log-rank test; *P* < 0.001 for XFM cells). The median survival time of the virus-treated mice injected with NP53 cells was 48.5 days, in comparison with 28 days for the controls (Figure 4B). Moreover, Delta-24-ACT treatment showed significantly better efficacy than Delta-24-RGD or radiotherapy (the standard treatment) in the NP53 model (log-rank test; *P* = 0.001 Supplemental Figure 3, A and B). In addition, 25% (2 of 8) of the NP53-bearing mice were long-term survivors (Figure 4B). We also evaluated the antitumor efficacy of Delta-24-ACT in the XFM DIPG model using the same schedule (Figure 4E). The survival time was 30 days for the treated group of mice injected with XFM cells compared with 9 days for the controls. Importantly, 50% (5 of 10) of the XFM-bearing mice were long-term survivors (Figure 4E).

Because DIPGs are known to recur after treatment, and the goal of this project was not only to show local efficacy, but also to achieve protective immunological memory, we performed a rechallenge experiment with long-term survivors of the NP53 experiment. All of the naive mice used as controls (4 of 4) developed tumors, while Delta-24-ACT–treated mice showed 100% protection against rechallenge with NP53 cells (2 of 2; Figure 4C). To demonstrate that the antitumor response observed was due to the immune response, we performed an experiment in immunodeficient (BALB/cA-Rag2^−/−^γc^−/−^) mice bearing NP53 murine cells, as the replication of the virus is highly attenuated in murine cell lines. Treatment with the virus did not present a survival benefit in this model (*P* = 0.622; Figure 4D). Next, we assessed the antitumor effect of viral treatment in already established XFM tumors, and we postponed viral treatment until 7 days, instead of 3 days, after cell injection (Figure 4F). Delta-24-ACT significantly increased the median survival of treated mice and led to a 30% increase in the long-term survival rate (3 of 10; *P* = 0.0093 Figure 4G). Moreover, 66% of the treated XFM tumor-bearing mice exhibited immunological memory (2 of 3; Figure 4H), and these mice were free of tumors (Figure 4I). Thus, Delta-24-ACT treatment is efficacious against DIPG murine models and induces protective immunological memory against local tumor rechallenge.

### Delta-24-ACT triggered a proinflammatory response in DIPG models.

**Figure 5:**
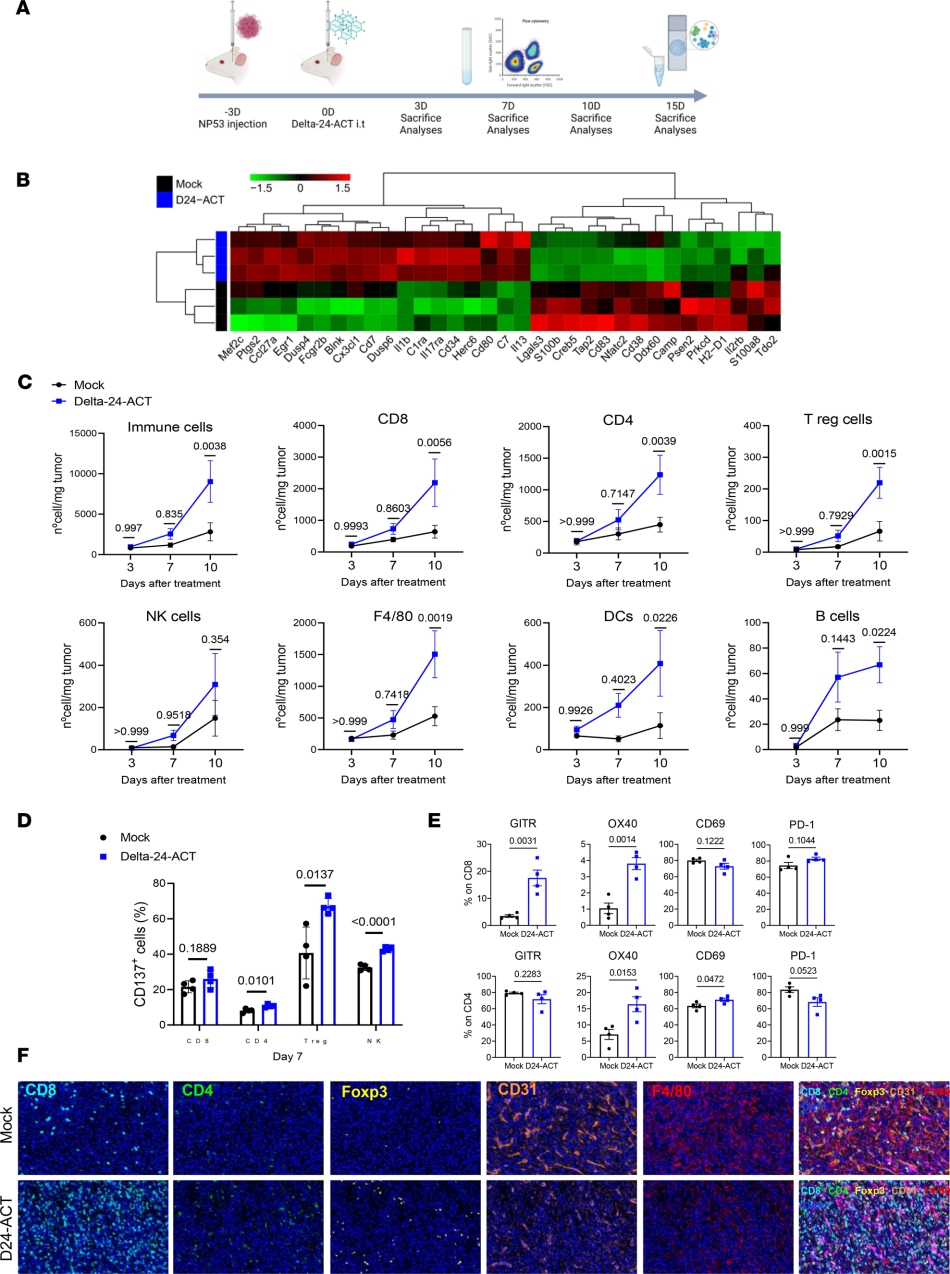
Modulation of the tumor microenvironment by Delta-24-ACT treatment. (**A**) Schedule of mechanistic studies in the NP53 model. NP53 cells were engrafted (day –3), and animals were treated with a mock control or Delta-24-ACT (10^6^ PFUs) 3 days later. Animals were sacrificed 3 (3D), 7 (7D) or 10 (10D) days later for flow cytometry and 15 (15D) days later for NanoString and IHC multiplex analyses. (**B**) Representative heatmap of transcriptome profiling using gene set enrichment analysis of murine DIPG tumors from mock-treated and Delta-24-ACT–treated mice (*n* = 3) using the 770-gene pancancer immunoprofile panel in NanoString. (**C**) Flow cytometry analyses of different immune cell populations in the brains of mice bearing NP53 tumors on the indicated days after treatment with Delta-24-ACT (blue) or PBS (black). Data are shown as number of cells/mg tumor. Two-way ANOVA was performed, and *P* values are shown above respective bars. (**D**) CD137 expression (percentage) in T cell populations and NK cells 7 days after viral treatment. Multiple comparisons *t* test was performed (*n* = 4 each group), and *P* values are shown above respective bars. Data are shown as the mean ± SEM. (**E**) Flow cytometry analyses of different activation (GITR, OX40, CD69) and exhaustion (PD-1) markers were performed in the CD8^+^ and CD4^+^ cell subsets at 7 days after viral administra^t^ion. Data are shown as the mean ± SEM (*n* = 4 each group), and *P* values are shown above respective bars. (**F**) The brains of mice bearing NP53 cells were subjected to multiplexed immunofluorescence analysis to detect the following immune cell markers: CD8 (light blue), CD4 (green), Foxp3 (yellow), CD31 (orange), F4/80 (red), and GFAP (pink). The nuclei were counterstained with DAPI (blue). Representative micrograph are shown (*n* = 3) Original magnification, ×20.

To better understand the mechanism underlying the therapeutic effect of Delta-24-ACT, we evaluated the triggered immune response. Thus, we intratumorally injected Delta-24-ACT or PBS (mock group) 3 days after NP53 cell implantation (on day 0). Then, tumor immune infiltrate was analyzed by NanoString and IHC multiplex (on day 15) and flow cytometry at 3, 7 and 10 days after viral administration (Figure 5A). Gene set enrichment analysis of the control- and Delta-24-ACT–treated tumors revealed an increase in positive regulators of the immune response related to the activation and proliferation of lymphocytes (Figure 5B and Supplemental Figure 4, A and B). Similarly, flow cytometry analyses showed an increase over time in immune infiltration between the control- and Delta-24-ACT–treated groups. By day 10 after viral treatment, tumors were highly infiltrated by nearly all the populations tested, except for NK cells (CD45, *P =* 0.0038; CD8^+^, *P* = 0.0056; CD4^+^, *P* = 0.0039; Tregs, *P* = 0.0015; F4/80, *P* = 0.0019; B cells, *P* = 0.0224; NK cells, *P =* 0.354; DCs, *P* = 0.0226) (Figure 5C). We analyzed the expression of CD137 in CD8^+^, CD4^+^, Tregs (CD4^+^Foxp3^+^), and NK cells. On day 7, the 4 tumor-infiltrating populations had higher surface CD137 expression in the Delta-24-ACT–treated tumors than in the controls (Figure 5D) (CD8^+^, *P* = 0.1889; CD4^+^, *P* = 0.0101; Tregs, *P* = 0.0137; NK cells, *P* < 0.0001), indicating that they could directly interact with the cells expressing 4-1BBL in a Delta-24-ACT–dependent manner. This upregulation of CD137 expression was lost on day 10 (Supplemental Figure 5A). In addition to CD137, other activation/exhaustion markers, such as GITR, OX40, PD-1, and CD69, were analyzed in CD8^+^ and CD4^+^ T cells. We observed significantly upregulated expression of these markers in both populations on day 7 (Figure 5E) but not on day 10 (Supplemental Figure 5B). In addition, we analyzed tumor immune infiltration by immunohistochemistry 15 days after viral administration of NP53-bearing mice and observed a significant increase in the CD8^+^ subpopulation but no increase in CD4^+^ cells (*P* = 0.0168 and *P* = 0.5744, respectively). We also observed a significant increase in FOXP3^+^ Tregs after viral treatment (*P* = 0.048) (Supplemental Figure 5C). In parallel, we used multispectral immunofluorescence to confirm the localization of the CD8^+^, CD4^+^, FOXP3^+^, CD31^+^, and F4/80^+^ populations within the tumor 15 days after Delta-24-ACT treatment (Figure 5F and Supplemental Figure 5D). We first confirmed that immune cells could infiltrate the tumor, because the labeling was inside the tumor and not in the periphery. Moreover, we observed an increase in T populations in the Delta-24-ACT group, which was significant for Tregs (%CD8^+^, *P* = 0.1067; CD4^+^ density, *P* = 0.5422; Foxp3^+^ density, *P* = 0.0419). We also observed a decrease in the percentages of cells expressing the endothelial cell markers CD31 and F4/80 (which label microglia and macrophages), even though the differences were not significant (Figure 5F and Supplemental Figure 5D). Additionally, examination of long-term survivors from the rechallenge showed animals that were tumor free and lacking any kind of immune infiltration (Supplemental Figure 5E). Finally, we aimed to confirm these results in another DIPG tumor model (XFM), in this case adding Delta-24-RGD. Ten days after viral treatment (Supplemental Figure 5F), tumor-infiltrating lymphocytes (TILs) from XFM tumors were analyzed by flow cytometry. Similar to the results for NP53 tumors, we observed significant increases in the immune infiltrates in the Delta-24-ACT treatment group versus the control group (Supplemental Figure 5G). We observed major differences in the percentage of total immune cells (CD45^+^); this was due to the increase in the percentage of T lymphocytes, more specifically, to the CD8^+^ cytotoxic population. Although the proportions of CD4^+^ cells and Tregs tended to increase, their levels were not significantly different between the groups. We also did not observe any change in the percentage of NK cells between treatment groups (Supplemental Figure 5G). However, we wanted to see if there were differences in proliferation (Ki67 labeling) in the CD8^+^, CD4^+^, and NK populations due to the presence of the ligand (when comparing Delta-24-ACT with Delta-24-RGD and the control) (Supplemental Figure 5H). However, CD8^+^, CD4^+^, and NK cells present in Delta-24-ACT–treated tumors displayed significantly increased proliferation compared with Delta-24-RGD–treated or control tumors. Finally, we also observed that CD8^+^ and CD4^+^ lymphocytes expressed more PD-1 in the presence of Delta-24-ACT (Supplemental Figure 5H).

In order to elucidate whether genetic makeup could lead to differences in the immune infiltrate and, thus, in responses to the virus, we analyzed the basal tumor microenvironment in a spontaneous isogenic system. This murine DIPG model contains *TP53* and *PDGFR* mutations, and they differ in their histone 3 mutational status: H3-WT, H3-K27M, and H3-G34R. We assessed the percentage of different immune populations by flow cytometry in tumors presenting the above genetic mutations. Of importance, our analyses did not reveal significant differences among the 3 groups, except for in B cells and NKT cells, which were slightly higher in H3 WT group (Supplemental Figure 6, A and B). In addition, the tumor presented abundant microglia and macrophages (Supplemental Figure 6, A and B). In the same line, immunohistochemistry analysis of the brains from H3-WT and H3-K27M showed a nearly absent infiltration in both models (Supplemental Figure 7, A and B). Tumor infiltration, thus, was very similar in all genetic subgroups and comparable to that in the NP53 and XFM models.

In summary, altogether these data indicate that Delta-24-ACT can turn “cold” DIPG tumors into “hot” tumors by increasing the numbers of immune cells and their activation states, providing a solid rationale for the further translation of this virus into the clinic.

## Discussion

To date, DIPGs remain incurable. In this work, we built upon our previous preclinical and clinical experience with Delta-24-RGD to engineer a virus with superior efficacy. Our clinical studies on adult gliomas and DIPGs illustrate the use of oncolytic viruses and their efficacy in a subgroup of patients (17, 27, 28). Because we previously observed that treatment of DIPG immunocompetent models with the oncolytic virus Delta-24-RGD (15) resulted in increased TILs, strategies aimed at activating these lymphocytes seemed to be the next logical step. Several positive actionable immune checkpoints exist, including 4-1BB, OX40, CD27, and GITR (29). However, the broad spectrum of 4-1BB targets, including not only CD8 T lymphocytes, but also other important populations, such as NK cells (30, 31), prompted us to take advantage of its immune checkpoint ligands. More importantly, a 4-1BB agonist has been shown to exert antitumor effects in not only immunogenic tumor models, but also nonimmunogenic models; this is very relevant for DIPGs, because they are nearly devoid of infiltrating lymphocytes and present very few mutations (10–12). One important feature exhibited by 4-1BB agonists in preclinical studies is their capacity to disrupt immunological ignorance (32), which seems very important for tumors such as DIPGs, because their microenvironment is thought to be nonresponsive. In our model, Delta-24-ACT administration led to profound remodeling of the tumor microenvironment, leading to profuse tumor infiltration, which was most likely due to the virus itself and to the presentation of functional T cells due to the 4-1BBL. In fact, preclinical studies on different tumor models showed that 4-1BB agonist treatment resulted in restoration of T cell dysfunctionality in the tumor microenvironment (33) and increased persistence of tumor-specific T cells (34). For example, 4-1BB costimulation further enhanced the anti–PD-1–mediated reinvigoration of exhausted CD39^+^ CD8^+^ T cells from the primary and metastatic sites of epithelial ovarian cancers (35). We also observed an increase in Tregs in the NP53 model, probably as a result of the virus treatment, suggesting a potential resistance mechanism to this approach. However, examination of long-term survivors showed no trace of this population. Furthermore, studies will be needed to clarify the role of this cell compartment and the implications for virotherapy. In our study, 4-1BB was efficient at triggering an effective antitumor immune response and, more importantly, at establishing immunological memory. Interestingly, these results were different from those in our study in glioma models, in which this virus was not able to trigger immune memory when long-term survivors were subjected to rechallenge (22). These results suggest a different mechanism of action in DIPG models, which we know present a different microenvironment with less immunosuppression than adult gliomas (10, 11). Unfortunately, although 4-1BB agonists were very effective in preclinical models, clinical trials assessing efficacy were hampered by high toxicity, specifically in the liver (reviewed in ref. 20). However, vectorizing 4-1BBL into a virus could circumvent all the toxicities associated with the systemic administration of 4-1BB agonists while maintaining a safe profile. Supporting this notion, we found no hepatic toxicity. Moreover, an Ankara virus armed with the 4-1BBL as well as tumor-associated antigens, which was administered intratumorally was shown to be effective and safe for eradicating solid tumors (36).

Although oncolytic viruses have been extensively studied in the context of adult brain tumors, there is a paucity of studies that have addressed their suitability and efficacy for pediatric brain tumors and specifically for DIPGs. For example, one study showed that oncolytic herpesvirus 1716 inhibited the migration and invasion of pediatric high-grade glioma and DIPG cells (37); parvovirus H1 also exerted oncolytic effects on these DIPG cell lines in vitro (38). In addition to our study, other preclinical studies have addressed the therapeutic potential of oncolytic viruses; for example, the adenovirus CRAd.S.pK7 showed some degree of antitumor activity when loaded into mesenchymal stem cells (39). We previously demonstrated that Delta-24-RGD was effective at recruiting lymphocytes. More importantly, the virus appeared to be safe and exhibited modest therapeutic efficacy in preclinical models of DIPG (15). Of translational importance, our group began the first human clinical trial for the treatment of naive DIPG with Delta-24-RGD followed by radiotherapy (27). The results of this trial, although not yet published, support the feasibility, safety, and degree of efficacy of this virus in children with this devastating disease (NCT03178032; our unpublished observations). Importantly, another trial evaluating the effect of a virus based on the Delta-24 platform expressing the OX40 ligand for recurrent adult gliomas (DNX-2440; NTC03714334) is currently ongoing at our institution, and 11 patients have thus far been enrolled, with no relevant toxicities.

One limitation of this study is that murine models are not permissive to adenoviral replication (40); consequently, the antitumor effect we observed is hampered by the fact that the virus expresses 4-1BBL only once due to its inability to replicate in murine cells. Therefore, the effect we observed is somewhat suppressed at the therapeutic and safety levels. Nevertheless, our clinical study of DNX-2440 for recurrent gliomas has shown feasibility and safety, thus far supporting further translation to the clinic.

In summary, we herein provide robust evidence that the intratumoral delivery of Delta-24-ACT is capable of disrupting DIPG microenvironment tolerance by inducing profound proinflammatory changes, leading to the activation of T cells and to the generation of immune memory and, thus, preventing tumor recurrence. The results of this study, along with the excellent safety profile of the Delta-24 platform, provide a strong rationale for exploring this approach in the clinic.

## Methods

Further information can be found in Supplemental Methods.

### Research design.

The objective of this study was to determine the preclinical efficacy of Delta-24-ACT as a therapeutic approach for DIPG. After viral generation, 4-1BBL expression and functionality were assessed by qPCR, Western blotting, immunofluorescence, and flow cytometry assays. Granzyme B and IFN-γ were detected by flow cytometry and ELISA, respectively. MTS and replication assays were used to quantify the antitumor effects of Delta-24-ACT in vitro in DIPG human and murine cell lines. Evaluation of the in vivo antitumor effect and immune response to Delta-24-ACT was performed in immunocompetent mice bearing orthotopic tumors derived from NP53 or XFM murine DIPG cell lines. Histological analysis was performed to assess the localization of both the tumor and virus in vivo and other putative mechanisms involved in responses to viruses. All in vivo experiments were repeated at least 2 times unless mentioned otherwise. Immunophenotyping of the tumor microenvironment was performed via immunohistochemistry, multiplexed immunohistochemistry, and flow cytometry assays.

### Cell lines.

The murine NP53 and XFM cell lines were provided in-house. Both cell lines were obtained from DIPG tumors generated in genetically modified mice. NP53 cells were generated from a DIPG tumor driven by *PDGF-β* signaling, *p53* loss, and an *H3.3K27M* mutation (41), whereas XFM cells were generated from a tumor induced by PDGF-β signaling and *INK4A* and *ARF* loss (42). Both cell lines were maintained in DMEM supplemented with 10% fetal bovine serum and 1% antibiotics (streptomycin, penicillin).

The SU-DIPG IV cell line was provided by Michelle Monje (Standford University, Standford, California, USA), and TP54 was provided by Marie-Pierre Junier and Hervé Chneiweiss (INSERM Institute, Paris, France). Both human cell lines were maintained as neurospheres in serum-free specialized media. TP54 cells were cultured in medium supplemented with a human neural stem cell proliferation supplement (NeuroCult NS-A Proliferation Kit, 05751, STEMCELL Technologies) and basic fibroblast growth factor and epidermal growth factor (20 ng/mL; MilliporeSigma), while SU-DIPG IV cells were cultured in DMEM/F12 supplemented with B27 (17504-044, Gibco), heparin, and basic fibroblast growth factor and epidermal growth factor (20 ng/mL). The HEK293 (CRL-1573, ATCC) and A549 (CCL-185, ATCC) cell lines were used for viral construction. All cell lines were maintained in a humidified atmosphere at 37°C and 5% CO_2_.

### Delta-24-ACT construction.

Delta-24-ACT was constructed by maintaining the Delta-24-RGD modifications of a 24–base pair deletion and introduction of RGD; however, *m4-1BBL* was incorporated in the E3 locus after removing this gene. Briefly, murine *4-1BBL* was first cloned into a pCDNA3.1 plasmid using the *KpnI* and *XhoI* restriction enzymes (New England Biolabs). Then, *m4-1BBL*, flanked with the cytomegalovirus promoter and bovine growth hormone polyadenylation sequences, was subcloned into the pAB26-RGD plasmid (24) at the *ClaI*/*BamHI* site. Finally, the 4-1BBL expression cassette was introduced into pVK-500C-24, and the Delta-24 plasmid was constructed by homologous recombination with pAB26-m4-1BBL in BJ5183 bacteria. For viral rescue, the obtained plasmid was linearized with *Pac I* and transfected into HEK293 cells with Lipofectamine 2000 (Invitrogen). After confirmation of genetic modifications by PCR and sequencing, Delta-24-ACT was amplified in A549 cells, purified, and stored at –80°C.

### Immunoblotting.

Cell lysates were obtained by treating the samples with lysis buffer (1X PBS + 1% Triton X-100) together with a protease inhibitor for 30 minutes on ice and centrifuging for 20 minutes at 4°C. The protein amount was assessed using a Bradford colorimetric assay, and 30 μg protein was subjected to sodium dodecyl sulfate-tris-glycine gel electrophoresis and then transferred to nitrocellulose membranes, which were incubated with the following antibodies: E1A (1:1000, Sc-430, Santa Cruz Biotechnology), adenovirus fiber (1:1000, NB600-541, Novus Biologicals), 4-1BBL (1:1000, AF1246, R&D Systems), and GRB2 (1:1000, 610112, BD). Finally, the membranes were developed according to the Amersham enhanced chemiluminescence protocol.

### NanoString gene expression analysis.

RNA was isolated from FFPE tumor sections by dewaxing using deparaffinization solution (QIAGEN). Total RNA was extracted using the RecoverAll Total Nucleic Acid Isolation Kit (Ambion) according to the manufacturer’s instructions. RNA purity was assessed on an ND NanoDrop 1000 spectrometer (Thermo Fisher Scientific). For the NanoString platform, 100 ng RNA was used to detect immune gene expression using the nCounterPanCancer Immune Profiling Panel along with custom CodeSet. Counts of the reporter probes were tabulated for each sample by the nCounter Digital Analyzer, and the raw data output was imported into R/Bioconductor (43). First, gene expression data were normalized with the NACHO (44) R package. After quality assessment and outlier detection using R/Bioconductor, a filtering process was performed. Genes without read counts in more than 50% of the samples of all the studied conditions were considered not expressed in the experiment under study. LIMMA (Linear Models for Microarray Data) (45) was used to identify the genes with significant differential expression between experimental conditions. Genes were selected as differentially expressed using a *P* value cutoff of *P* < 0.01. Further functional and clustering analyses were performed and graphical representations were generated using clusterProfiler (46) and R/Bioconductor. The nanostring data from this study have been submitted to the Gene Expression Omnibus under accession GSE197374.

### Flow cytometry.

For 4-1BBL expression analysis, 1.5 × 10^4^ cells/well were infected with Delta-24-ACT (MOIs of 25 and 50 for murine cells and 5 and 10 for human cells) and harvested 48 hours later for staining with a PE-coupled anti-4-1BBL antibody (1:200, 107105, Biolegend). Dead cells were removed by Zombie NIR staining (1:1000, 423105, Biolegend), and the remaining samples were then analyzed using the FACSCanto II system (BD Biosciences).

For TIL analysis, brains were collected, and tumor samples were disaggregated to obtain a cell suspension. Briefly, the tumor samples were disaggregated mechanically and then chemically with collagenase and DNase I before being passed through a 40 μm cell strainer. Finally, the obtained suspension was subjected to 30% Percoll treatment to obtain TILs. Cell suspensions were incubated with 100 μL of the antibodies resuspended in PBS 1X + 0.5% FBS + 0.5% EDTA. The antibodies used are listed in Table 1. Dead cells were removed by PromoFluor-840 staining (1:10,000, PK-PF840-3-01, PromoCell), and the remaining samples were then analyzed using CytoFLEX (Beckman Coulter) and FACSDiva software (BD Biosciences).

### Immunofluorescence.

NP53 and XFM cells (5 × 10^5^) were seeded on glass slides and infected with Delta-24-ACT at an MOI of 50 for 24 hours (1 group was not infected as a control). Forty-eight hours after infection, the cells were fixed with 4% formaldehyde/methanol-free (28906, Thermo Fisher Scientific) and blocked for 1 hour at room temperature (S0809, Dako). Finally, the cells were stained with a PE-coupled anti-4-1BBL antibody (1:200, 107105, Biolegend) for 2 hours at room temperature, and the nuclei were stained with DAPI.

### Immunohistochemical analysis.

Paraffin-embedded mouse brain sections were first stained with hematoxylin and eosin for tumor analysis. Once tumor status was confirmed, the remaining staining experiments were performed with antibodies against adenovirus rabbit E1A (1:1000; Santa Cruz Biotechnology), CD3 (1:300; clone SP7, NeoMarkers), CD4 (1:1000; EPR19514, ab183685, Abcam), CD8α (1:1000, [D4W2Z], 98941, Cell Signaling), and FoxP3 (1:400; clone JFK-16s, ref 14–5773, eBiosciences, Thermo Fisher Scientific). For immunohistochemical staining, Vectastain ABC kits (Vector Laboratories Inc.) were used according to the manufacturer’s instructions. The number of positively stained cells per mm^2^ was quantified using the Fiji platform.

### Multiplexed immunofluorescence.

A multiplex immunolabeling protocol based on tyramide signal amplification (TSA) and Opal fluorophores was developed and validated as previously described (47). Briefly, paraffin-embedded sections of mouse brains were deparaffinized, hydrated, and treated with peroxidase. Then, each section was subjected to sequential rounds of antibody staining, each including heat-induced antigen retrieval at pH 6 and protein blocking with 20% normal goat serum (Dako) in PBS. Finally, the sections were incubated with a primary antibody and secondary antibody HRP conjugate (Dako), followed by TSA visualization with fluorophores Opal 520, Opal 540, Opal 570, Opal 620, Opal 650, and Opal 690 (Akoya Biosciences).

The primary antibodies included CD8 (rabbit monoclonal, 98941, 1:500, Cell Signaling Technology), CD4 (rabbit monoclonal, 25229, 1:400, Cell Signaling Technology), CD31 (rabbit monoclonal, 77699, 1:400, Cell Signaling Technology), FOXP3 (rabbit monoclonal, 12653, 1:600, Cell Signaling Technology), GFAP (rabbit monoclonal, 80788, 1:100, Cell Signaling Technology), and F4/80 (rabbit monoclonal, 70076, 1:400, Cell Signaling Technology). After 6 sequential reactions, nuclei were counterstained with spectral DAPI (Akoya Biosciences), and sections were mounted with Diamond antifade mountant (Life Technologies).

Multiplexed immunofluorescence slides were scanned on a Vectra-Polaris Automated Quantitative Pathology Imaging System (Akoya Biosciences) as previously described (47, 48). The whole tissue present in a single FFPE tissue section was imaged, spectrally unmixed, and exported as a component TIF image tile using Akoya Biosciences Inform software (version 2.4.8). Component TIF image tiles were then imported into the open source digital pathology software QuPath version 0.2.0-m9 and stitched together using the *x*-*y* coordinates to create a new pyramidal TIF file.

### Assessment of CD8^+^ lymphocyte activation.

CD8^+^ cells from B6. CgThy1a-Tg(TcraTcrb)Rest/J (PMEL) mice were isolated with a CD8a^+^ T cell isolation kit (130-104-075, Miltenyi Biotec) according to the manufacturer’s instructions and seeded with CD3 (clone 145-2C11, 100314 Biolegend) and CD28 (clone 37.51, 102112 Biolegend) antibodies for 24 hours. NP53 cells were infected with Delta-24-RGD, Delta-24-ACT, or a mock control at an MOI of 100 for 48 hours and then incubated with 1 μg/mL hgp100 (RP20344, GenScript) for 2 hours (mock NP53 and NP53-ACT cells without hgp100 served as controls). Finally, CD8^+^ cells and target cells were cocultured for 48 hours, and the cells and supernatants were then collected. IFN-γ expression was analyzed in the supernatants using a mouse IFN-γ DuoSet ELISA kit (DY485, R&D Systems) according to the manufacturer’s instructions. Cells were stained with the intracellular granzyme-B antibody (25-8898-80, Thermo Fisher Scientific) for flow cytometry assessment.

### Animal studies.

10^4^ NP53 cells were injected into the pons of transgenic mice provided in-house, and 10^3^ XFM cells were injected into the pons of female BALB/c mice using a screw-guided system (26). Three days after cell injection, 2 μL PBS or Delta-24-ACT (10^6^ PFUs/mouse) was administered intratumorally. Animals that showed obvious symptoms of disease were sacrificed, and survival curves were plotted according to the Kaplan-Meier method.

### Serum biochemistry.

We obtained serum samples from NP53-bearing mice 3 days after Delta-24-ACT administration (or PBS in the control group), and serum transaminase, ALP, bilirubin, and albumin levels were measured using a Cobas C311 Autoanalyzer (Roche).

### Statistics.

The in vitro experiments were repeated at least 3 times. Dose-response curves for viral cytotoxicity were obtained by nonlinear regression. Data with normal distributions were assessed by Shapiro-Wilk tests, and comparisons among groups were performed with 2-tailed nonparametric tests with 95% CIs for nonnormally distributed data sets or parametric tests when normality was confirmed (2-tailed Student’s *t* test or 1- or 2-way ANOVA). For the comparison of groups in survival experiments, a log-rank test (Mantel-Cox) was used. GraphPad Prism 8 (Statistical Software for Sciences) was used for the statistical analyses. *P* values less than 0.05 were considered significant.

### Study approval.

Ethical approval for the animal studies was granted by the Animal Ethical Committee of the University of Navarra under protocol 094-15. All animal studies were performed at the veterinary facilities of the Center for Applied Medical Research in accordance with institutional, regional, and national laws and ethical guidelines for experimental animal care.

## Author contributions

VL, MMA, JF, and CGM conceived and designed this study. VL, MP, SL, LM, MGM, MZ, MGH, NMV, IAM, DDLN, GHC, JMS, EG, CEDA, MV, OB, MS, VM, JGPL, APG, SG, CGM, JF, and MMA developed methodology; acquired data (provided animals, acquired and managed patients, provided facilities, etc.); analyzed and interpreted the data (e.g., statistical analysis, biostatistics, and computational analysis); wrote, reviewed, and/or revised the manuscript; and provided administrative, technical, or material support (i.e., reporting or organizing data and constructing databases). MMA, JF, and CGM supervised the study.

## Supplementary Material

Supplemental data

## Figures and Tables

**Table 1:**
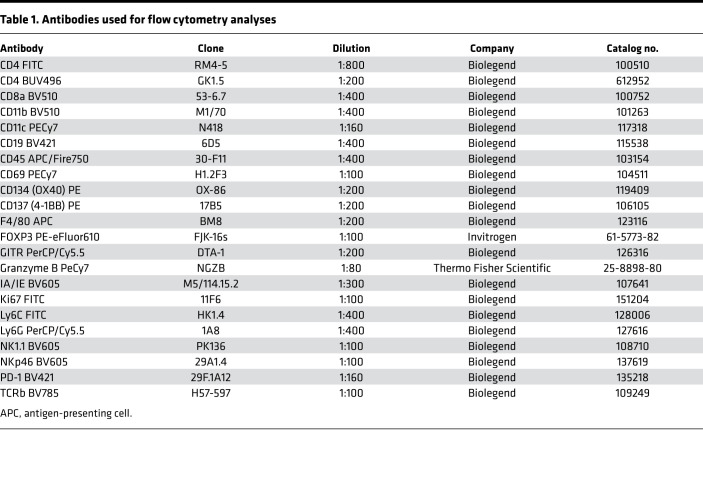
Antibodies used for flow cytometry analyses

## Version 1 04/08/2022

Electronic publication

## References

[1] Cooney T (2017). Contemporary survival endpoints: an international diffuse intrinsic pontine glioma registry study. Neuro Oncol.

[2] Hoffman LM (2017). Clinical, radiologic, pathologic, and molecular characteristics of long-term survivors of diffuse intrinsic pontine glioma (DIPG): a collaborative report from the International and European Society for pediatric oncology DIPG registries. J Clin Oncol.

[3] Grasso CS (2015). Functionally defined therapeutic targets in diffuse intrinsic pontine glioma. Nat Med.

[4] Lowe BR (2019). Histone H3 mutations: an updated view of their role in chromatin deregulation and cancer. Cancers (Basel).

[5] Hoeman CM (2019). ACVR1 R206H cooperates with H3.1K27M in promoting diffuse intrinsic pontine glioma pathogenesis. Nat Commun.

[6] Cordero FJ (2017). Histone H3.3K27M represses p16 to accelerate gliomagenesis in a murine model of DIPG. Mol Cancer Res.

[7] Panditharatna E (2015). Clinicopathology of diffuse intrinsic pontine glioma and its redefined genomic and epigenomic landscape. Cancer Genet.

[8] El-Khouly FE (2019). Diagnostics and treatment of diffuse intrinsic pontine glioma: where do we stand?. J Neurooncol.

[9] Vanan MI, Eisenstat DD (2015). DIPG in children — what can we learn from the past?. Front Oncol.

[10] Lieberman NAP (2019). Characterization of the immune microenvironment of diffuse intrinsic pontine glioma: implications for development of immunotherapy. Neuro Oncol.

[11] Lin GL (2018). Non-inflammatory tumor microenvironment of diffuse intrinsic pontine glioma. Acta Neuropathol Commun.

[12] Ross JL (2021). Platelet-derived growth factor beta is a potent inflammatory driver in paediatric high-grade glioma. Brain.

[13] Chen DS, Mellman I (2017). Elements of cancer immunity and the cancer-immune set point. Nature.

[14] Bommareddy PK (2018). Integrating oncolytic viruses in combination cancer immunotherapy. Nat Rev Immunol.

[15] Martínez-Vélez N (2019). The oncolytic virus Delta-24-RGD elicits an antitumor effect in pediatric glioma and DIPG mouse models. Nat Commun.

[16] Martinez-Velez N (2019). Delta-24-RGD combined with radiotherapy exerts a potent antitumor effect in diffuse intrinsic pontine glioma and pediatric high grade glioma models. Acta Neuropathol Commun.

[17] Lang FF (2018). Phase I study of DNX-2401 (Delta-24-RGD) oncolytic adenovirus: replication and immunotherapeutic effects in recurrent malignant glioma. J Clin Oncol.

[18] Wang C (2009). Immune regulation by 4-1BB and 4-1BBL: complexities and challenges. Immunol Rev.

[19] Croft M (2003). Co-stimulatory members of the TNFR family: keys to effective T-cell immunity?. Nat Rev Immunol.

[20] Chester C (2018). Immunotherapy targeting 4-1BB: mechanistic rationale, clinical results, and future strategies. Blood.

[21] Yonezawa A (2015). Boosting cancer immunotherapy with anti-CD137 antibody therapy. Clin Cancer Res.

[22] Puigdelloses M (2021). CD137 and PD-L1 targeting with immunovirotherapy induces a potent and durable antitumor immune response in glioblastoma models. J Immunother Cancer.

[23] Fueyo J (2000). A mutant oncolytic adenovirus targeting the Rb pathway produces anti-glioma effect in vivo. Oncogene.

[24] Fueyo J (2003). Preclinical characterization of the antiglioma activity of a tropism-enhanced adenovirus targeted to the retinoblastoma pathway. J Natl Cancer Inst.

[25] Fucikova J (2020). Detection of immunogenic cell death and its relevance for cancer therapy. Cell Death Dis.

[26] Obeid M (2007). Calreticulin exposure dictates the immunogenicity of cancer cell death. Nat Med.

[27] Marigil M (2017). Development of a DIPG orthotopic model in mice using an implantable guide-screw system. PLoS One.

[28] Tejada S (2017). Phase I trial of DNX-2401 for diffuse intrinsic pontine glioma newly diagnosed in pediatric patients. Neurosurgery.

[29] Tejada S (2018). DNX-2401, an oncolytic virus, for the treatment of newly diagnosed diffuse intrinsic pontine gliomas: a case report. Front Oncol.

[30] Mellman I (2011). Cancer immunotherapy comes of age. Nature.

[31] Morvan MG, Lanier LL (2016). NK cells and cancer: you can teach innate cells new tricks. Nat Rev Cancer.

[32] Chiossone L (2018). Natural killer cells and other innate lymphoid cells in cancer. Nat Rev Immunol.

[33] Wilcox RA (2002). Provision of antigen and CD137 signaling breaks immunological ignorance, promoting regression of poorly immunogenic tumors. J Clin Invest.

[34] Williams JB (2017). The EGR2 targets LAG-3 and 4-1BB describe and regulate dysfunctional antigen-specific CD8+ T cells in the tumor microenvironment. J Exp Med.

[35] Weigelin B (2015). Focusing and sustaining the antitumor CTL effector killer response by agonist anti-CD137 mAb. Proc Natl Acad Sci U S A.

[36] Leem G (2020). 4-1BB co-stimulation further enhances anti-PD-1-mediated reinvigoration of exhausted CD39^+^ CD8 T cells from primary and metastatic sites of epithelial ovarian cancers. J Immunother Cancer.

[37] Hinterberger M (2021). Intratumoral virotherapy with 4-1BBL armed modified vaccinia Ankara eradicates solid tumors and promotes protective immune memory. J Immunother Cancer.

[38] Cockle JV (2017). Oncolytic herpes simplex virus inhibits pediatric brain tumor migration and invasion. Mol Ther Oncolytics.

[39] Josupeit R (2016). Pediatric and adult high-grade glioma stem cell culture models are permissive to lytic infection with parvovirus H-1. Viruses.

[40] Chastkofsky MI (2020). Mesenchymal stem cells successfully deliver oncolytic virotherapy to diffuse intrinsic pontine glioma. Clin Cancer Res.

[41] Halvorson KG (2015). A high-throughput in vitro drug screen in a genetically engineered mouse model of diffuse intrinsic pontine glioma identifies BMS–754807 as a promising therapeutic agent. PLoS One.

[42] Barton KL (2013). PD-0332991, a CDK4/6 inhibitor, significantly prolongs survival in a genetically engineered mouse model of brainstem glioma. PLoS One.

[43] Gentleman RC (2004). Bioconductor: open software development for computational biology and bioinformatics. Genome Biol.

[44] Canouil M (2020). NACHO: an R package for quality control of NanoString nCounter data. Bioinformatics.

[45] Ritchie ME (2015). limma powers differential expression analyses for RNA-sequencing and microarray studies. Nucleic Acids Res.

[46] Yu G (2012). clusterProfiler: an R package for comparing biological themes among gene clusters. OMICS.

[47] Abengozar-Muela M (2020). Diverse immune environments in human lung tuberculosis granulomas assessed by quantitative multiplexed immunofluorescence. Mod Pathol.

[48] Martinez-Valbuena I (2019). Amylin as a potential link between type 2 diabetes and Alzheimer disease. Ann Neurol.

