# Supplementary material for: Exploiting 4-1BB immune checkpoint to enhance the efficacy of oncolytic virotherapy for diffuse intrinsic pontine gliomas
Source: JCI Insight. 2022 Apr 8;7(7):e154812. doi: 10.1172/jci.insight.154812 (PMC9057625; doi:10.1172/jci.insight.154812)
Supplement: Supplemental data [file jciinsight-7-154812-s178.pdf]

Supplementary Materials:

Supplementary material and methods

Cell viability assay

All the cell lines were seeded in 96-well plates (NP53 and XFM at a density of 500 cell/well and SU-DIPG IV and TP54 at $1x10^4$ cell/well). Then, the cells were infected with Delta-24-ACT at MOIs ranging from 5 to 100. Five days after infection, cell viability was assessed using the CellTiter 96® Aqueous One Solution Cell Proliferation Assay (Promega, Fitchburg, WI, USA; G3581) as previously described *(46)*. Viability curves were analyzed using GraphPad Prism 8 (Statistical Software for Sciences) to determine the IC_{50} values of Delta-24-ACT in the tested cell lines (IC_{50} is the viral dose at which 50% of cells are affected, i.e., 50% survival).

qRT-PCR

RNA was extracted with TRIzol at 48 h after cell infection ($1.5x10^4$ cells/well) with Delta-24-ACT at MOIs of 50 and 100. Then, cDNA was obtained from 1 µg of RNA by RT-PCR, and gene expression was analyzed by real-time PCR using Fast SYBR Green PCR Master Mix (Applied Biosystems).The specific primers were as follows:

4-1BBL Fw: 5´CTGTGTTCGCCAAGCTACTG3´ and Rv: 5´GGGACTGTCTACCACCAACT3´; $GAPDH_{mouse}$Fw: 5´GGGAAATTCAACGGCACAGT3´ and Rv: 5´AGATGGTGATGGGCTTCCC3´; and $GAPDH_{human}$Fw: 5´AGCCACATCGCTCAGACAC3´ and Rv: 5´GCCCAATACGACCAAATCC3´.

Measurement of DAMPs

Each cell line was plated at a density of $2x10^5$ cells per well (six-well plates) and was infected with Delta-24-ACT at its corresponding three-day IC_{50}. At 72 h later, the concentrations of the DAMP markers—HMGB1 (ST51011; IBL International), and ATP (ENLITEN® ATP Assay System; FF2000; Promega)—were measured in supernatants of infected and mock-infected cultures.

Immunoblotting

Cell lysates were obtained by treating the samples with lysis buffer (PBS 1X + 1% Triton X-100) together with a protease inhibitor for 30 minutes on ice and centrifuging for 20 minutes at 4°C. The protein amount was assessed using a Bradford colorimetric assay (BCA), and 30 µg of protein were subjected to sodium dodecyl sulfate-tris-glycine gel electrophoresis and then transferred to nitrocellulose membranes, which were incubated with the following antibodies: E1A (1:1000, Sc-430 Santa Cruz Biotechnology), fiber (1:1000, NB600-541 Novus Biologicals), 4-1BBL (1:1000, AF1246 R&D Systems) and GRB2 (1:1000, 610112 BD). Finally, the membranes were developed according to the Amersham enhanced chemiluminescence protocol.

Mice for the isogenic system

All injections were performed in mouse pups aged 0–2 days old among equally distributed sexes for each genotype. *Ntva*;*Ptenfl*/*fl*;*Cdkn2a*−/−mice are in a mixed genetic background while *Ntva*;*Ptenfl*/*fl* mice are in a pure C57BL/6 background (Herting *et al.*, 2017). Mice were housed in a climate-controlled, pathogen-free facility with access to food and water *ad libitum* under a 12-h light/dark cycle. *Ccl2* (#004434) and *Ccl7* (#017638) knockout mice were obtained from the Jackson Laboratory and crossed to C57BL/6 *Ntva*;*Ptenfl*/*fl* mice until homozygous knockout mice were obtained as previously described for the generation of *Ntva*;*Ptenfl*/*fl*;*Ccl2*−/− mice (Tsou *et al.*, 2007;

Chen *et al.*, 2017). *Ccl3* knockout mice were gifted by Dr C. K. Qu, *Ccl8*/*12*−/− mice were gifted by Dr Sabina Islam, and both were crossed with C57BL/6 *Ntva*;*Ptenfl*/*fl* mice until homozygous knockout mice were obtained. Genotyping of all mice was performed using Transnetyx. All experimental procedures were approved by the Institutional Animal Care and Use Committee of Emory University (Protocol #2003253, #201700633) and Icahn School of Medicine at Mount Sinai (Protocol #201900619).

Cell culture and mice injections

DF1 cells (ATCC) were grown at 39°C, expanded to passage 4 and transfected with RCAS-PDGFB-HA, RCAS-shp53-RFP, RCAS-H3.3WT, RCAS-H3.G34R or RCAS-H3.3K27M using a FuGENE® 6 Transfection kit FuGENE 6 Transfection reagent (Promega, Cat. E2691), accordingly to manufacturer's protocol. Cells were cultured with DMEM media (Gibco, 11995-065) supplemented with 1× l-glutamine, 1× penicillin/streptomycin, and 10% fetal bovine serum (FBS) (ATCC). Media containing $2x10^5$ DF1 cells transfected with the different construction was injected in the IV ventricle of 0-2 days old *GFAP-Tva; GFAP-Cre; Rosa26-LSL-Cas9; p53lox/lox* mice using a 30-gauge needle attached to a Hamilton gas-tight syringe (Fisher Scientific,#14-815-250). Mice were closely monitored for signs of tumor development and sacrificed when reaching humane end-point. At this time point, mouse brains were extracted for further analysis.

Immunephenotyping of the isogenic tumors by flow cytometry

After sacrificing, mice were perfused with ice-cold PBS and whole brains obtained for processing. Tumors were digested mechanically and enzymatically with 0.4 mg/ml collagenase (R8757, Merck) and DNAseA (10104159001, Roche) in RPMI + 10% FBS. Erythrocytes were lysed in presence of RBC lysis buffer (00-4333-57, Labclinics) for 2

minutes at room temperature. Then, samples underwent 22% Percoll (17-0891-01, GE Healthcare) gradient separation to obtain TILs.

Cell suspensions were first stained with LIVE/DEAD Fixable Blue Dead Cell Stain (L34962, Life technologies) to differentiate non-viable cells, and then with 100 µl of the antibody mix. The used antibodies are listed in the table below (table 2). For FOXP3 staining cells were fixed in True-Nuclear™ fix reagent (424401, Biolegend) and then permeabilized with True-Nuclear™ permeabilization buffer (424401, Biolegend). Data was acquired using a LRS Fortessa cytometer.

Table 2. List of the antibodies used for the flow cytometry experiment.

ANTIBODY	CLONE	DILUTION	COMPANY	REFERENCE
CD45 PECy7	30-F11	1:400	Invitrogen	25-0451-82
CD3 APC	145-2C11	1:100	BD Pharmingen	553066
NK1.1 BV711	PK136	1:100	Biolegend	108745
B220 BUV661	RA3-6B2	1:100	BD Biosciences	612972
CD11b PerCP-Cy5.5	M1/70	1:100	Invitrogen	45-0112-82
F4/80 APC/Fire750	BM8	1:50	Biolegend	123152
CD204 PE	M204PA	1:200	Invitrogen	12-2046-82
CD4 BV510	RM4-5	1:200	BD Biosciences	563106
CD8 FITC	53-6.7	1:400	Tonbo Biosciences	35-0081
CD25 PerCP-Cy5.5	PC61	1:100	Biolegend	102030
FOXP3 PE	MF-14	1:40	Biolegend	126404

Supplementary Figures:

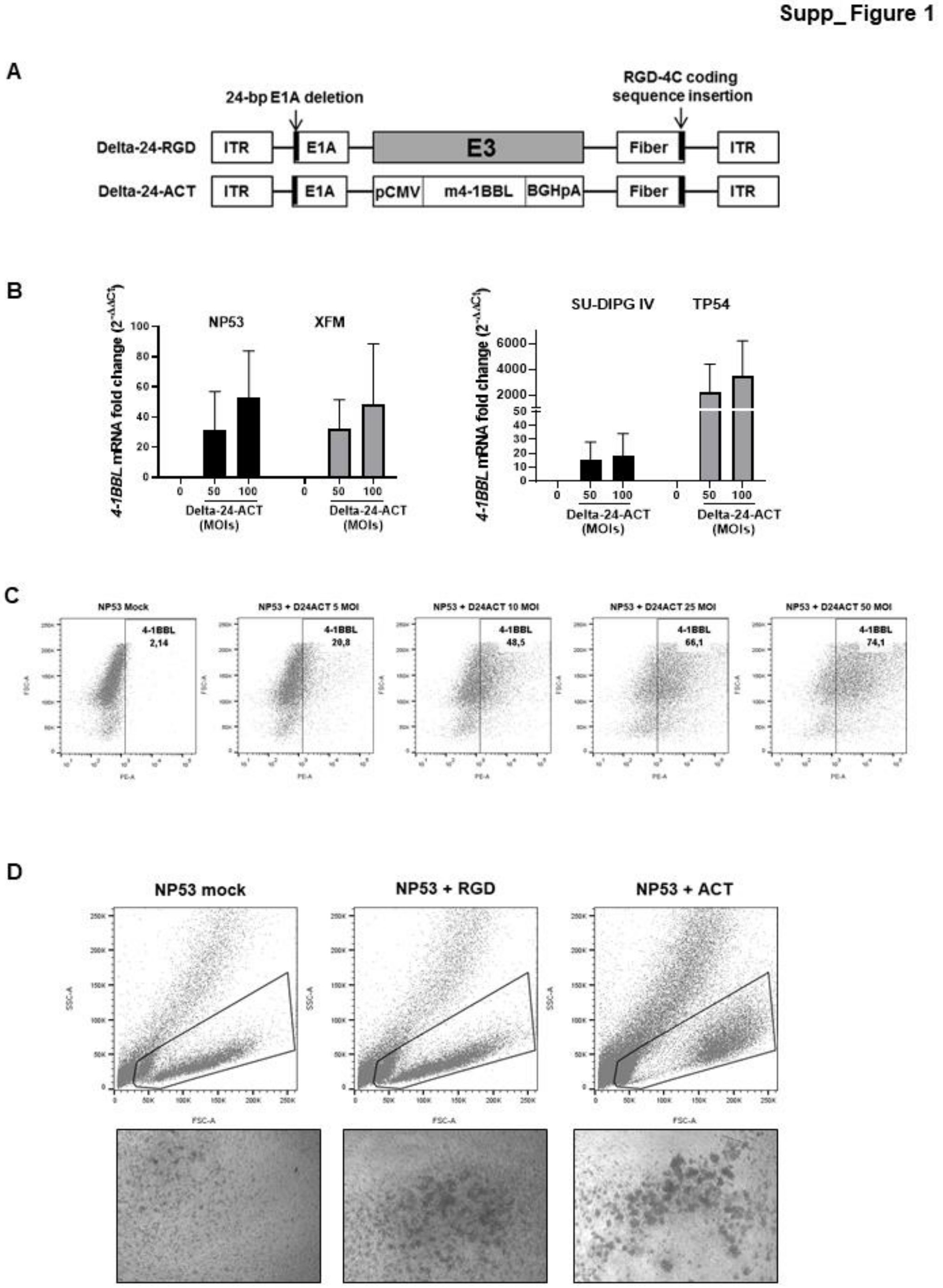

Supplementary figure 1. The 4-1BBL expression in DIPG cells after Delta-24-ACT infection. (A) Schematic representation of the engineered Delta-24-ACT. **(B)** The m4-1BBL expression as determined by qPCR in NP53 and XFM murine cell lines and SU-DIPG IV and TP54 human cell lines after Delta-24-ACT infection at the indicated MOIs. **(C)** Representative images showing the 4-1BBL expression in the membranes of NP53 cells infected with Delta-24-ACT at different MOIs for 48 h as determined by flow cytometry. **(D)** ***Upper panel,*** representative flow cytometry image of CD8$^+$ lymphocytes cocultured with NP53 and NP53 cells infected with Delta-24-RGD or Delta-24-ACT (100 MOI). ***Lower panel,*** representative images of CD8$^+$ clusters acquired on an inverted microscope.

Supp_ Figure 2

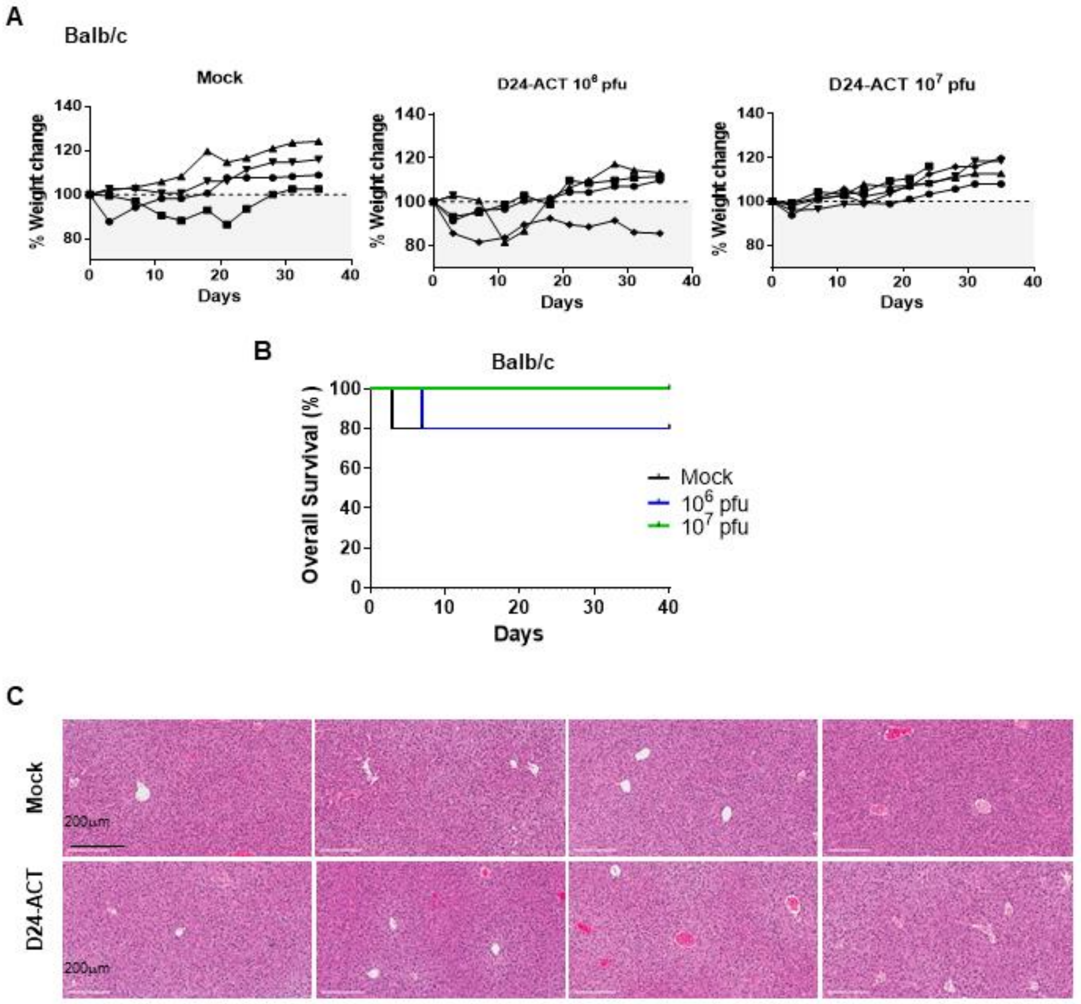

	Nº livers	Nº healthy livers
**Mock**	4	4 (100%)
**D24-ACT**	4	4 (100%)

Supplementary figure 2. Characterization of Delta-24-ACT toxicity in DIPG murine models. (A) Balb/c mice were treated intraparenchymally with mock (PBS) (*n*=5) or Delta-24-ACT (*n*=5) at the indicated doses. Mice from the different groups were weighed every 3-4 days until the end of the treatment (30 days). **(B)** Kaplan-Meier survival plot of Balb/c mice treated with PBS (control group) and 10^6 PFUs or 10^7 PFUs of Delta-24-ACT in the pons. **(C)** Representative histological images of the livers of mice bearing orthotopic DIPGs and treated locally with Delta-24-ACT at 10^8 PFUs. None of the livers analyzed showed hepatic injury.

Supp_ Figure 3

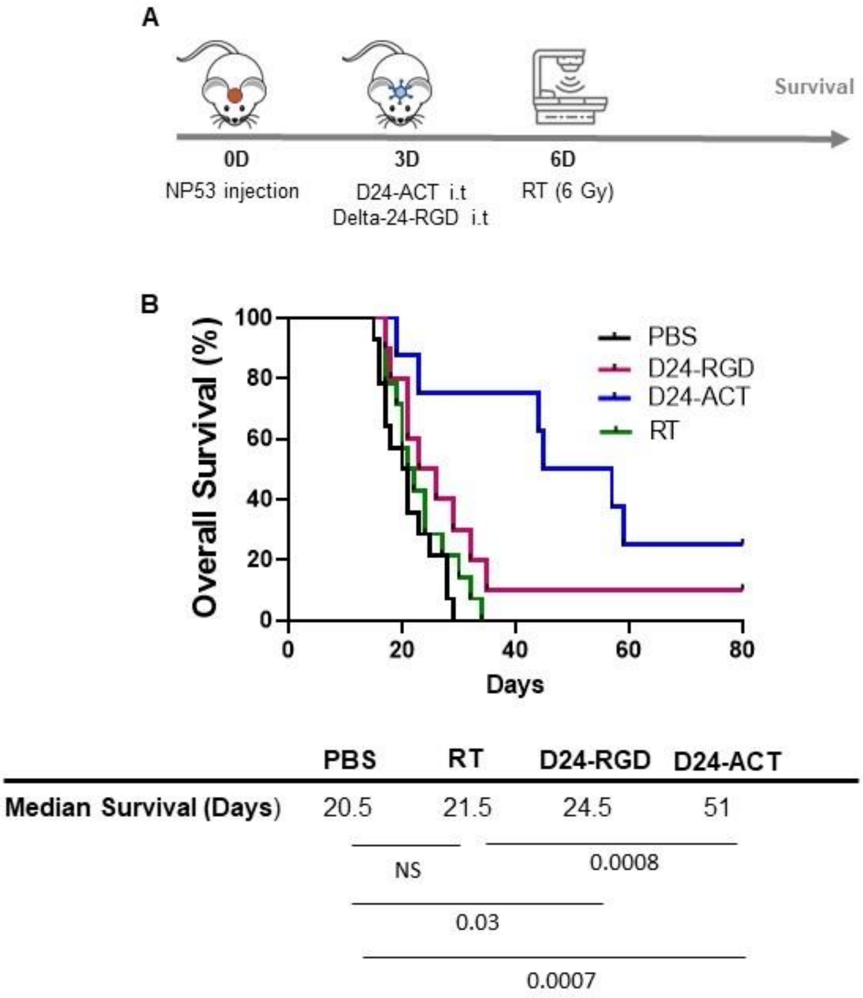

Supplementary figure 3. Characterization of the anti-tumor effect Delta-24-ACT in DIPG murine models. (**A**) Schedule of survival experiments performed with NP53 comparing the effect on survival of viruses Delta-24-RGD and Delta-24-ACT. **(B)** Kaplan-Meier survival plot of mice bearing NP53 cells treated with 10^6 PFUs of Delta-24-RGD or Delta-24-ACT or a mock PBS control (log-rank; *P=0.001*,PBS *n*=14; Delta-24-RGD *n*=10; Delta-24-ACT *n*=8).

Supp_ Figure 4

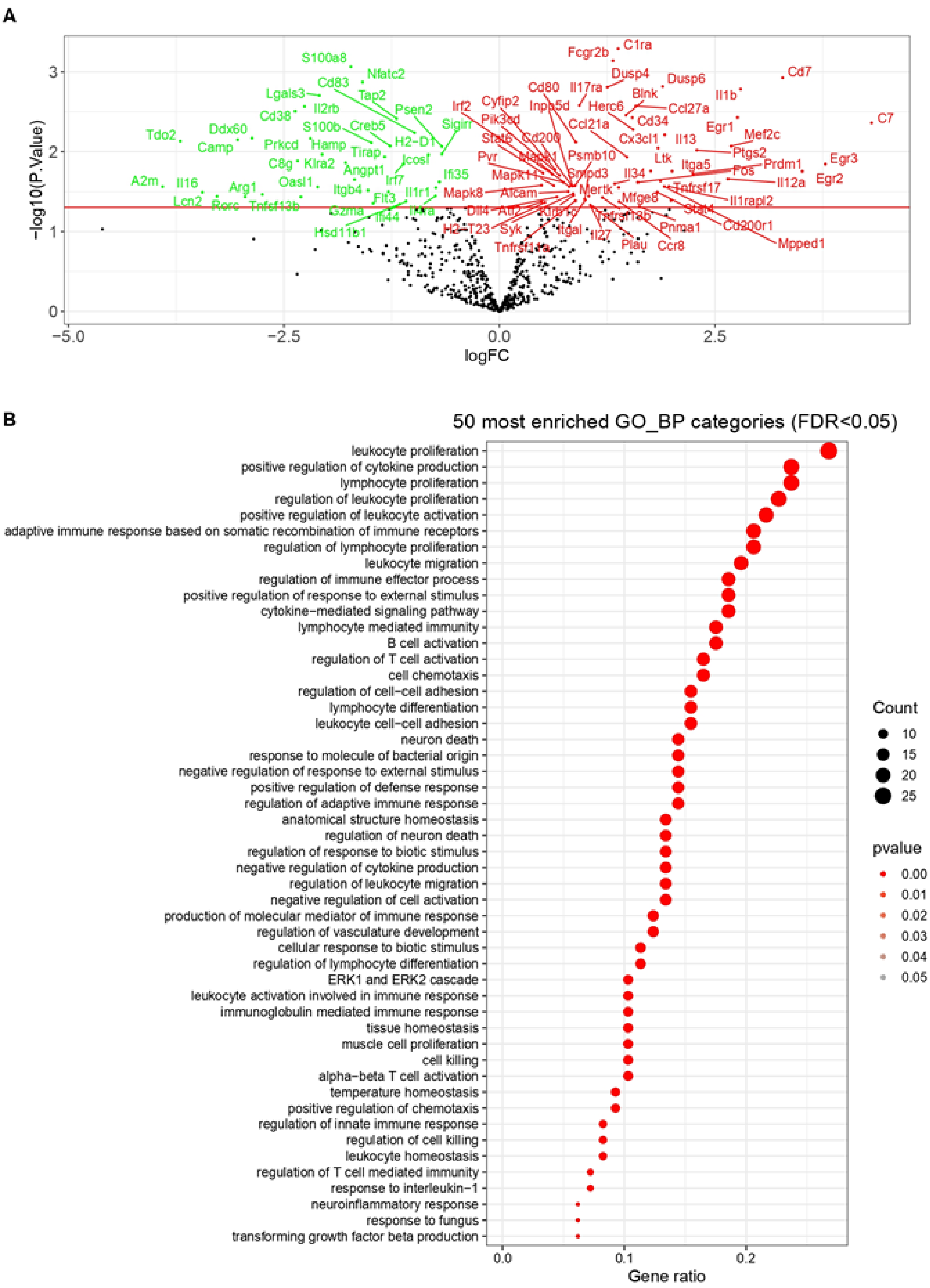

Supplementary figure 4. Evaluation of the tumor microenvironment after Delta-24-ACT treatment in the NP53 orthotopic model. (A) Volcano plot' of statistical significance against fold-change between control-treated and Delta-24-ACT-treated, demonstrating the most significantly differentially expressed genes. **(B)** Graph representing the 50 GO-biological processes enriched in Delta-24-ACT-treated animals versus control-treated.

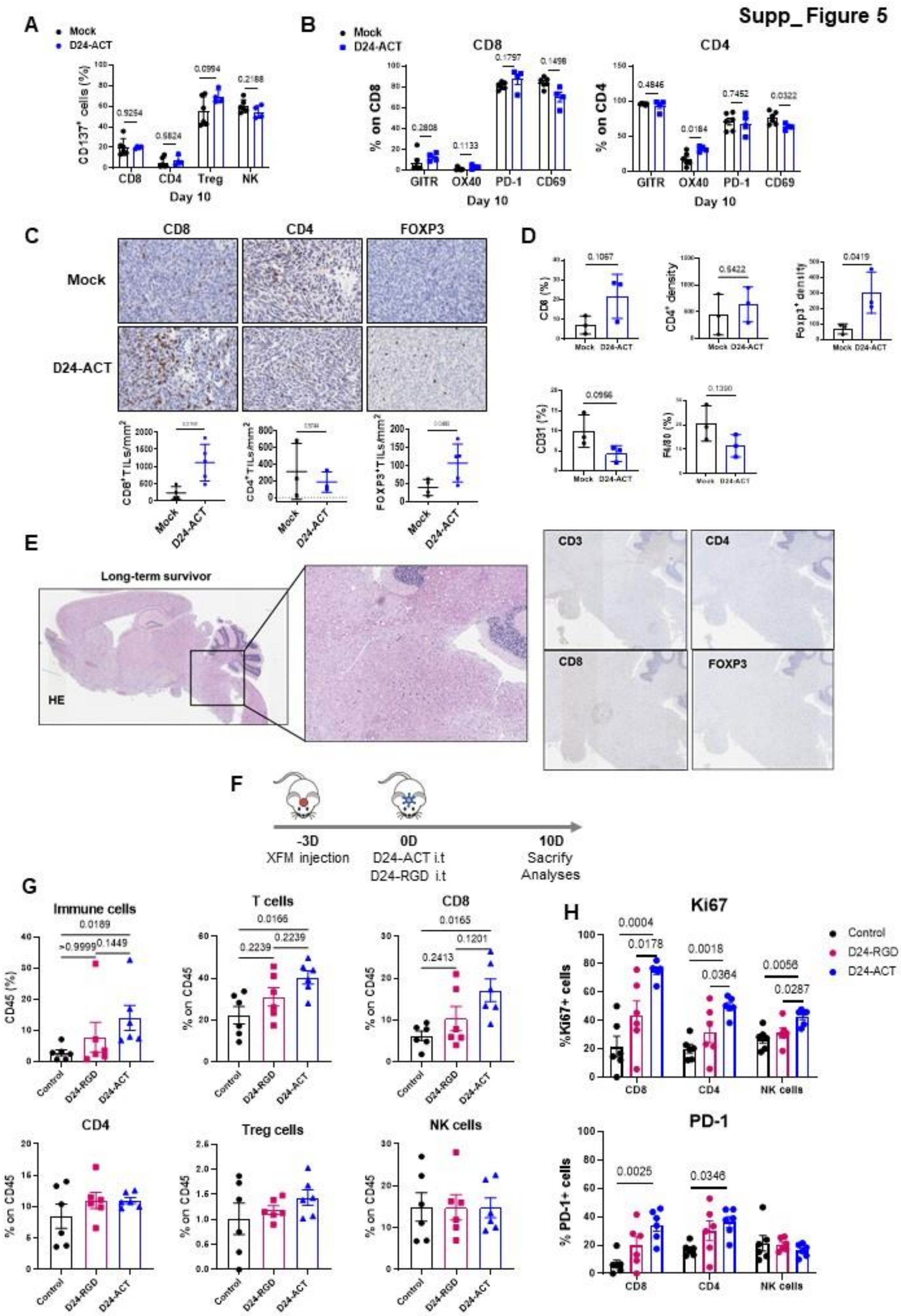

Supplementary figure 5. Modulation of the tumor microenvironment by Delta-24-ACT in the NP53 and XFM orthotopic models. (A) CD137 expression (%) in T cell populations and NK cells 10 days after viral treatment. Multiple t-test was performed (n=4/6) **(B)** Flow cytometry analyses of different activation (GITR, OX40, CD69) and exhaustion (PD-1) markers were performed on the CD4^{+} and CD8^{+} cell subsets 10 days after viral administration in NP53-bearing mice. The bars indicate the mean ± SD (n=6

PBS, *n*=4 D24-ACT) Multiple *t*-test. **(C)** ***Upper panel***, representative images (scale bar, 100 µm) of CD4, CD8, and FOXP3 immunostaining of DIPG tumors from control and Delta-24-ACT-treated mice. ***Lower panel***, quantification of positive $CD4^+$, $CD8^+$, and $FOXP3^+$ cell infiltration per mm^2 of DIPG tumors. Graph showing the quantification of positive cell infiltration at 15 days after cell implantation per mm^2 of tumors treated with either PBS or Delta-24-ACT (*n* =3-5). P values were calculated by the two-tailed Student's t test. **(D)** The brains of mice bearing NP53 cells were subjected to multiplexed immunofluorescence analysis to detectthe following immune cell markers: CD8 (light blue), CD4 (green), Foxp3 (yellow), CD31 (orange), F4/80 (red), and GFAP (pink). Nuclei were counterstained with DAPI (blue). Quantification of the different immune populations at 15 days after treatment (*n*=3). **(E)** Representative micrographs of a NP53 long-term survivor showing H&E and CD3, CD4, CD8, and FOXP3 immunostaining of DIPG tumors from Delta-24-ACT-treated mice subjected to a rechallenge. **(F)** Schedule of mechanistic studies in the XFM model. XFM cells were engrafted (day -3), and the animals were treated with either a mock control, Delta-24-ACT or Delta-24-RGD (10^6 PFUs) three days later. The animals were sacrificedten days (10D) later, and the different immune populations were assessed. **(G)** Flow cytometry analyses of different immune cell populations in the brains of mice bearing XFM tumors 10 days after treatment with Delta-24-ACT (blue), Delta-24-RGD (magenta) or PBS (black). The bars indicate the mean ± SD (*n*=6), One-way ANOVA. **(H)** Flow cytometry analyses of Ki67 proliferation and PD-1 exhaustion markers were performed in the CD8, CD4 and NK cell subsets 10 days after viral administration. The bars indicate the mean ± SD (*n*=6).

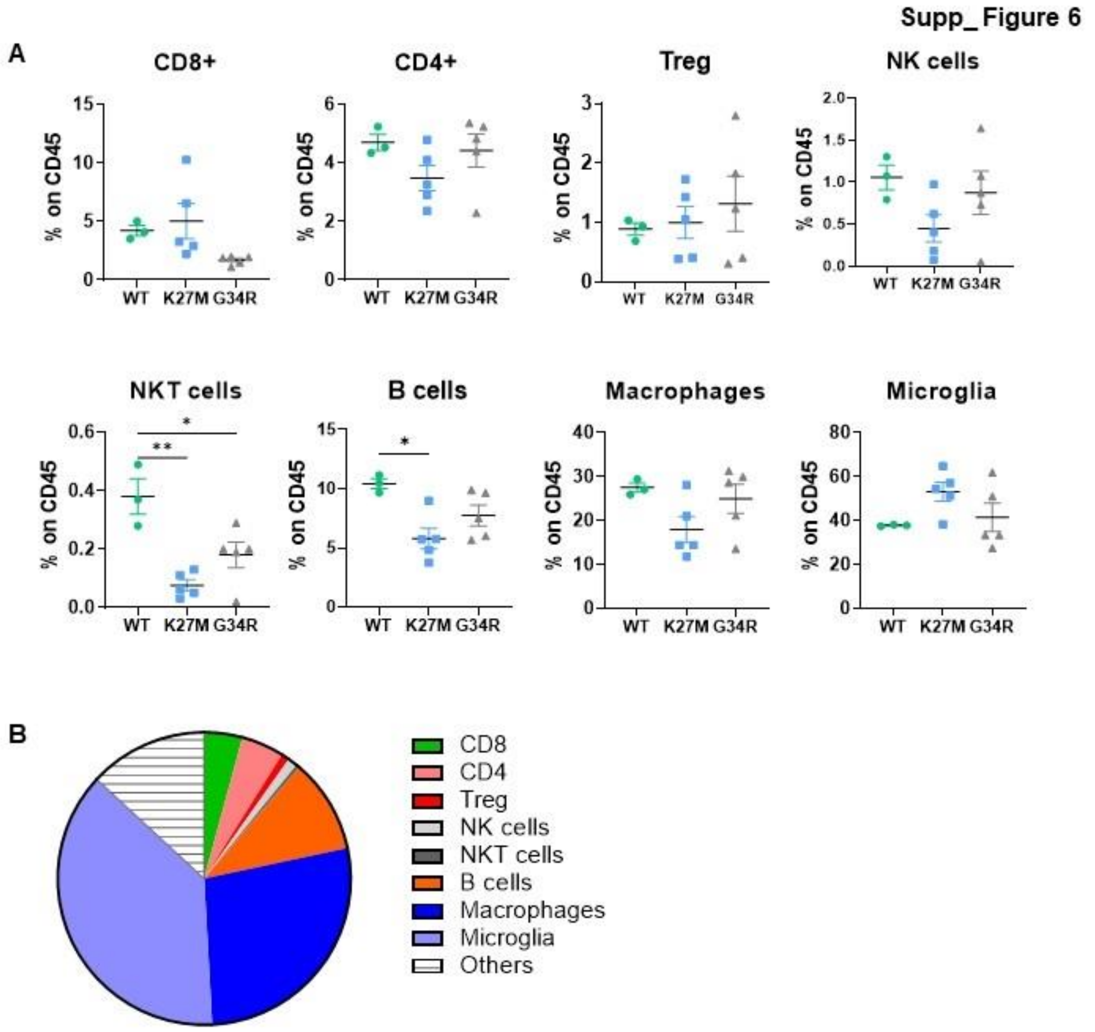

Supplementary Figure 6. Analyses of the composition of TME in an isogenic model. (A) Flow cytometry analyses of different immune populations of tumors in an isogenic system. Cells contain TP53 and PDGFR mutations in addition to H3 WT, H3K27M or H3G34R mutations. Data are shown as percentage (%) on CD45+ immune cells. One-way ANOVA was performed (N_{WT}=3, $N_{K27M,G34R}$=5). **(B)** Schematic representation of the proportions of immune cell populations forming the TME. The median percentages of the individuals of H3 WT group are shown.

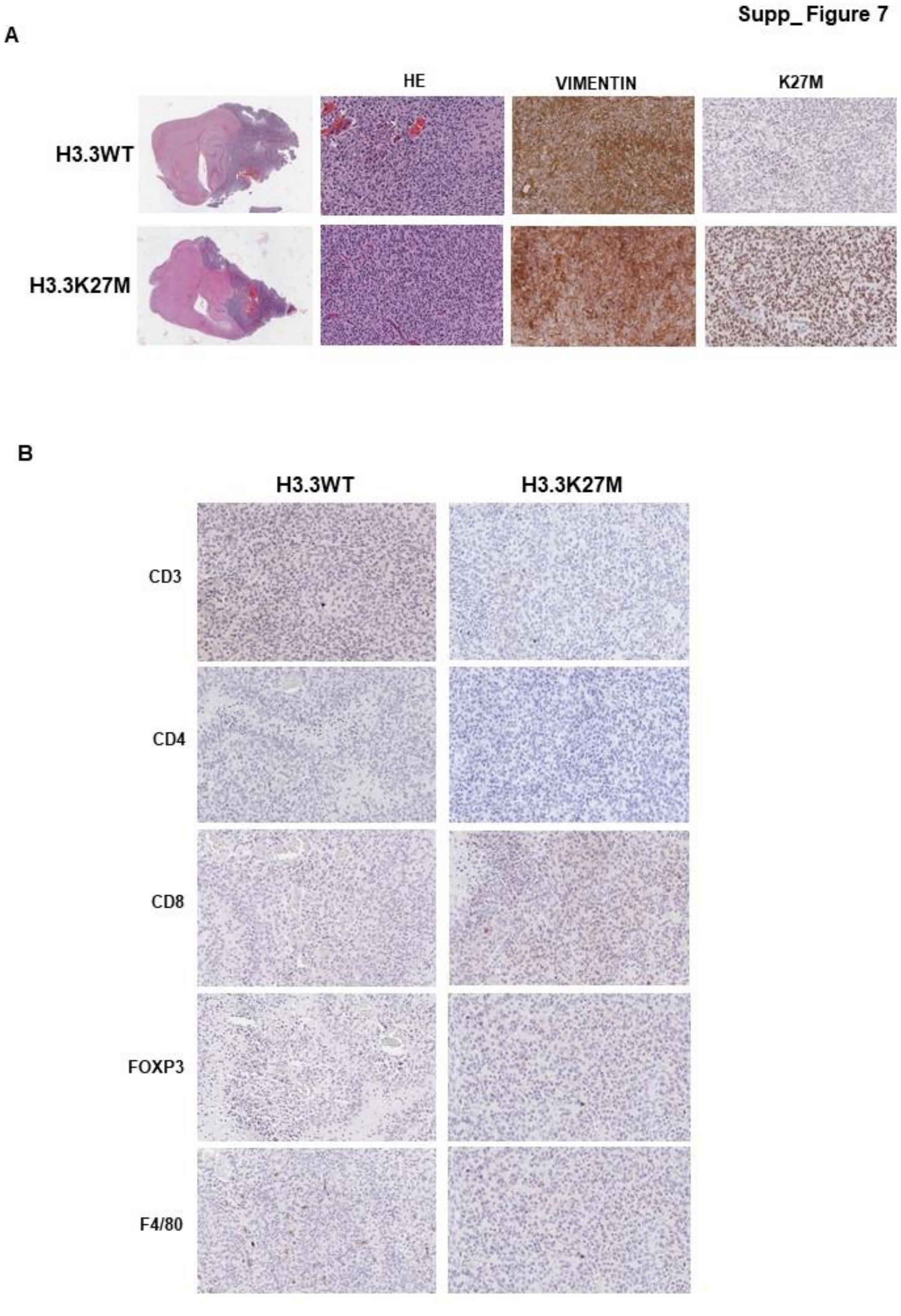

Supplementary Figure 7. Analyses of the composition of TME in an isogenic model. (A) Representative micrographs (100 µm) of spontaneous tumors arising from H3 WT or H3K27M transgenic mice. Showing H&E and H3 mutations and vimentin immunostaining. **(B)** Representative micrographs of spontaneous tumors arising from H3 WT or H3K27M transgenic mice. Showing CD3, CD4, CD8, FOXP3 and F4/80 immunostaining.
